# Lightweight Dual-Functional Segregated Nanocomposite Foams for Integrated Infrared Stealth and Absorption-Dominant Electromagnetic Interference Shielding

**DOI:** 10.1007/s40820-024-01450-0

**Published:** 2024-06-17

**Authors:** Zhonglei Ma, Ruochu Jiang, Jiayao Jing, Songlei Kang, Li Ma, Kefan Zhang, Junxian Li, Yu Zhang, Jianbin Qin, Shuhuan Yun, Guangcheng Zhang

**Affiliations:** 1 MOE Key Laboratory of Material Physics and Chemistry Under Extraordinary Conditions, Shaanxi Key Laboratory of Macromolecular Science and Technology, School of Chemistry and Chemical Engineering, Northwestern Polytechnical University, Xi’an, 710072 People’s Republic of China; 2 College of Chemistry and Chemical Engineering, Key Laboratory of Auxiliary Chemistry and Technology for Chemical Industry, Ministry of Education, Shaanxi University of Science and Technology, Xi’an, 710072 People’s Republic of China; 3 Department of Mechanical and Industrial Engineering, University of Toronto, 5 King’s College Road, Toronto, ON M5S 3G8 Canada; 4 Chongqing Innovation Center, Northwestern Polytechnical University, Chongqing, 401135 People’s Republic of China

**Keywords:** Segregated nanocomposite foams, Microcellular structures, Infrared stealth, EMI shielding, Low infrared emissivity

## Abstract

Lightweight infrared stealth and absorption-dominant electromagnetic interference (EMI) shielding materials are highly desirable in areas of aerospace, weapons, military and wearable electronics. Herein, lightweight and high-efficiency dual-functional segregated nanocomposite foams with microcellular structures are developed for integrated infrared stealth and absorption-dominant EMI shielding via the efficient and scalable supercritical CO_2_ (SC-CO_2_) foaming combined with hydrogen bonding assembly and compression molding strategy. The obtained lightweight segregated nanocomposite foams exhibit superior infrared stealth performances benefitting from the synergistic effect of highly effective thermal insulation and low infrared emissivity, and outstanding absorption-dominant EMI shielding performances attributed to the synchronous construction of microcellular structures and segregated structures. Particularly, the segregated nanocomposite foams present a large radiation temperature reduction of 70.2 °C at the object temperature of 100 °C, and a significantly improved EM wave absorptivity/reflectivity (*A*/*R*) ratio of 2.15 at an ultralow Ti_3_C_2_T_*x*_ content of 1.7 vol%. Moreover, the segregated nanocomposite foams exhibit outstanding working reliability and stability upon dynamic compression cycles. The results demonstrate that the lightweight and high-efficiency dual-functional segregated nanocomposite foams have excellent potentials for infrared stealth and absorption-dominant EMI shielding applications in aerospace, weapons, military and wearable electronics.

**Supplementary Information:**

The online version contains supplementary material available at 10.1007/s40820-024-01450-0.

## Highlights

- Lightweight dual-functional segregated nanocomposite foams are developed via the supercritical CO_2_ (SC-CO_2_) foaming combined with hydrogen bonding assembly and compression molding strategy
- The segregated nanocomposite foams exhibit superior infrared stealth performances benefitting from the synergistic effect of highly effective thermal insulation and low infrared emissivity.
- Excellent absorption-dominant electromagnetic interference shielding performances are achieved owing to the synchronous construction of microcellular structures and segregated structures

**Supplementary Information:**

The online version contains supplementary material available at 10.1007/s40820-024-01450-0.

## Graphical abstract

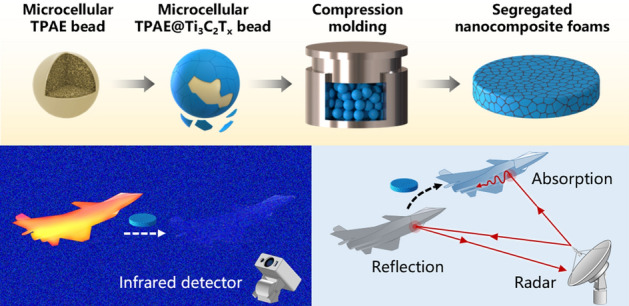

## Introduction

With the prosperity of modern electronic devices and wireless telecommunication, the information leakage and electromagnetic pollution caused by infrared target exposure (such as temperature changes generated by operation of electronics and fighter engines) and electromagnetic interference (EMI) are becoming increasingly serious in areas of aerospace, weapons, military and wearable electronics [1–6]. They detrimentally effect the information security and operational reliability of precision electronics [7–10]. Therefore, high-efficiency infrared stealth and EMI shielding materials are tremendously desired to protect the infrared target and attenuate the electromagnetic (EM) waves [11–14]. Metals, such as aluminum, copper and silver, are typical low infrared emissivity and high EMI shielding materials. However, they display serious disadvantages including heavy weight, difficult processing and high cost. The conductive polymer composites (CPCs) with conductive fillers dispersed in polymer matrix have been investigated for lightweight EMI shielding [15–18]. Nevertheless, high filler contents are needed to achieve the satisfied electrical conductivity and EMI shielding performances, resulting in the weakened mechanical properties and processability [19–23]. Moreover, the reflection-dominant EMI shielding mechanism of metals and CPCs due to the impedance mismatch between air and shields results in the secondary pollution of EM waves [24–26]. The radar stealth also requires the shields to attenuate EM waves via absorption in wide frequency range with low EM reflection. Therefore, it remains a significant challenge to develop lightweight and high-efficiency dual-functional CPCs with integrated capacities of infrared stealth and absorption-dominant EMI shielding.

The introduction of cellular structures into CPCs offers high prospects in fabrication of lightweight polymer-based infrared stealth and EMI shielding composites [27–31]. According to Stefan–Boltzmann law, infrared stealth can be acquired by decreasing the infrared emissivity and/or reducing the surface temperature of protected targets [32, 33]. The cellular structures can not only decrease the mass density of CPCs for lightweight purposes, but also reduce the surface temperatures for infrared stealth based on their thermal insulation features [34, 35]. Xu et al. [36] fabricated the lightweight and thermally insulating PEDOT:PSS@melamine (PPM) foams for infrared stealth with a decreased infrared emissivity of 0.757. The PPM foams covered on the hot stage (80 °C) present a decreased radiation temperature of 44.1 °C with a temperature reduction (∆*T*) of 35.9 °C. Besides, the cell growth process can promote the formation of effective conductive networks via orientation of conductive fillers, leading to the enhanced multiple internal reflections of EM waves and total EMI shielding efficiency (SE) [37–39]. Moreover, the cellular structures can improve the impedance matching between air and shields and decrease the direct reflection of EM waves on the surfaces, leading to the absorption-dominant EMI shielding behaviors [40–43]. Several approaches such as chemical foaming [44], freeze‑drying [45], sacrificial template [46], 3D printing [40, 47] and supercritical carbon dioxide (SC-CO_2_) foaming [48] can been used for the fabrication of cellular CPCs. Among these, the SC-CO_2_ foaming process resembles an environmentally friendly, low-cost and efficient physical-blowing technique with gentle critical conditions (*T*_c_ = 31.3 °C and *P*_c_ = 7.38 MPa) for the fabrication of microcellular foams with cell sizes less than 100 μm and large cell densities [49, 50]. Park et al. [51] reported the significantly improved absorptivity/reflectivity (*A*/*R*) ratio by introducing microcellular structures in the polyvinylidene fluoride/carbon nanotube/SiC nanowire (PVDF/CNT/SiCnw) composites. The obtained microcellular composite foams show absorption-dominated EMI shielding performances with an EMI SE of 22 dB in Ku-band and an *A*/*R* ratio of 1.07 (higher than 1.0). Zhang et al. [52] fabricated the microcellular Ni-chain/PVDF foams with an EMI SE of 26.8 dB and specific SE (SSE) of 127.62 dB cm^2^ g^−1^ in X band by SC-CO_2_ foaming. Benefitting from the microcellular structures and Ni-chain conductive-magnetic networks, the microcellular foams exhibit absorption-dominant EMI shielding performances. Nevertheless, the improvement of infrared stealth and EMI shielding performances by single introduction of cellular structures is limited and is insufficient to meet the rigorous demands in high-tech applications.

Constructing conductive segregated structures in CPCs is demonstrated to be an effective strategy to fabricate polymer-based EMI shielding composites with significantly decreased percolation threshold at ultralow filler contents [53–55]. The conductive fillers present a selective distribution at the interfaces of neighboring polymer microdomains to form highly efficient conductive networks, resulting in the improved electrical properties and enhanced EMI shielding performances [56–58]. The multiple internal reflection of EM waves within segregated structures also can improve the EMI SE via absorption [59–61]. Recently, Yan et al. [62] successfully prepared the flexible interface-reinforced segregated carbon nanotube/polydimethylsiloxane (CNT/PDMS) composite with a high EMI SE of 47.0 dB at the CNT content of 2.2 vol% and a high tensile strength of 3.6 MPa. Wang et al. [63] constructed the segregated structures in biodegradable porous multi-walled carbon nanotube/polylactic acid (MWCNT/PLA) composites, achieving the enhanced thermal insulation and absorption-dominant EMI shielding performances. Ma et al. [64] reported the preparation of highly resilient segregated MWCNT/PDMS nanocomposites-based piezoresistive sensors for human motion detection by incorporating the silver coated microcellular thermoplastic polyether-block-amide elastomer (TPAE) beads, which contain the crystalline polyamide hard segments and polyether soft segments in the molecule chains. The microcellular TPAE beads with lightweight, high flexibility and resilience exhibit great potentials in aerospace, weapons, military and wearable electronics. The results provide new strategies for the development of lightweight and high-efficiency polymer-based infrared stealth and EMI shielding materials.

In this work, we report the lightweight and high-efficiency dual-functional segregated microcellular TPAE beads coated with Ti_3_C_2_T_*x*_ (TPAE@Ti_3_C_2_T_*x*_) nanocomposite foams for infrared stealth and absorption-dominant EMI shielding via the efficient and scalable SC-CO_2_ foaming combined with hydrogen bonding assembly and compression molding. Benefitting from the synergistic effect of highly effective thermal insulation and low infrared emissivity, the segregated nanocomposite foams exhibit outstanding infrared stealth performances. The synchronous construction of microcellular structures and segregated structures endows the segregated nanocomposite foams with lightweight and absorption-dominant EMI shielding performances at low Ti_3_C_2_T_*x*_ contents. Moreover, the segregated nanocomposite foams exhibit outstanding infrared stealth and EMI shielding stability upon dynamic compression cycles. The convenient and low-cost strategy endows the segregated nanocomposite foams with great prospect of large-scale fabrication. The influences of microcellular TPAE expansion ratio and Ti_3_C_2_T_*x*_ content on the microstructures, mechanical and electrical properties as well as the infrared stealth and EMI shielding performances have been investigated in detail. The lightweight and high-efficiency dual-functional segregated nanocomposite foams with superior infrared stealth and absorption-dominant shielding performances have promising application potentials in aerospace, weapons, military and wearable electronics.

## Experimental Section

### Materials

Thermoplastic polyamide elastomer (TPAE) beads (shore D hardness: 35, mass density: 1.01 g cm^−3^) were provided by Arkema Inc. Ti_3_AlC_2_ (MAX) powders (200 mesh) were obtained from Laizhou Kai Kai Ceramic Materials Co., Ltd. CO_2_ gas with a 99.99% purity was utilized as the physically foaming agent. Other chemicals including lithium fluoride (LiF), hydrochloric acid (HCl, 37 wt%) and formic acid (AC) were supplied by Sinopharm Chemical Reagent Co., Ltd.

### Preparation of Microcellular TPAE Beads

Microcellular TPAE beads were prepared by the environmentally friendly solid-state SC-CO_2_ foaming process. The solid TPAE beads were firstly placed in the autoclave filled with SC-CO_2_ at 45 °C and 15 MPa for 5 h, achieving the saturated gas concentration of 135 mg CO_2_ per gram of TPAE (Fig. S1). After pressure releasing, the saturated TPAE beads were transferred into the preheated kettle at 125 °C under uniform mechanical stirring for microcellular foaming. The microcellular TPAE beads with different expansion ratios (*β* = *ρ*/*ρ*_f_, where *ρ* and *ρ*_f_ are the mass densities of solid and microcellular TPAE beads, respectively) of 2.5, 4.2 and 5.5 were obtained with the foaming time of 25, 50 and 75 s, respectively.

### Synthesis of Ti_3_C_2_T_x_ MXene

The Ti_3_C_2_T_*x*_ MXene was obtained by chemical etching and delamination. 1.0 g of Ti_3_AlC_2_ was added to the etching solution consisting of 1.0 g LiF and 20 mL HCl solution with a concentration of 9 mol L^−1^. Etching was conducted at 35 °C upon magnetic stirring for 24 h, obtaining the accordion-like m-Ti_3_C_2_T_*x*_. The obtained dispersion was washed with deionized (DI) water by centrifuging at 3500 rpm for 5 min to reach a supernatant pH of approximately 6.0. Subsequently, the dispersion was sonicated at 180 W for 20 min and then centrifuged at 3500 rpm for 1 h to obtain the supernatant containing delaminated Ti_3_C_2_T_*x*_ MXene.

### Fabrication of Segregated Nanocomposite Foams

The microcellular TPAE beads with different expansion ratios were dip-coated in Ti_3_C_2_T_*x*_ dispersion to prepare the microcellular TPAE@Ti_3_C_2_T_*x*_ beads via hydrogen-bond assembly. After compression molding at 50 °C for 10 min in a cylindrical steel mold containing a small amount of formic acid, the TPAE@Ti_3_C_2_T_*x*_ beads were mutually bonded together by physical entanglement and hydrogen bonding interactions to obtain the lightweight and highly resilient segregated nanocomposite foams. The Ti_3_C_2_T_*x*_ content of obtained segregated nanocomposite foams was tailored by controlling the Ti_3_C_2_T_*x*_ dispersion concentrations during dip-coating (Table S1). The microcellular TPAE foams without Ti_3_C_2_T_*x*_ MXene were also prepared via compression molding for comparison.

### Characterizations

The morphologies of microcellular TPAE beads, microcellular TPAE@Ti_3_C_2_T_x_ beads, and segregated nanocomposite foams were assessed using a VEGA 3 LMH scanning electron microscope (SEM) with an energy-dispersive spectrometry (EDS). The samples were cut with a scalpel to reveal the fracture surfaces and sputter coated with Au/Pd. The microstructures of m-Ti_3_C_2_T_*x*_ and Ti_3_C_2_T_*x*_ MXene were observed with a FEI Verios 460 field emission SEM (FE-SEM) and a FEI Tecnai transmission electron microscope (TEM). The Image-Pro Plus software was applied to calculate the statistic cell-size distribution. The Archimedes water displacement method was employed to measure the mass densities. The Fourier-transform infrared spectroscopy (FTIR) analysis was conducted on a Thermo Nicolet spectrophotometer, and the X-ray photoelectron spectroscopy (XPS) analysis was performed on an Axis Ultra DLD spectrometer. The X-ray diffraction (XRD) patterns were obtained on a D8 AdvanceX diffractometer. The electrical conductivities of segregated nanocomposite foams were analyzed using the Princeton 4000 + electrometer. The cycling compression properties were conducted on the CMT8502 universal testing machine with a speed of 5 mm·min^−1^. The mid-infrared emissivity was obtained using a Nicolet iS50 FTIR spectrometer. The radiation temperatures and infrared images were obtained by a Fluke TiS75 + IR thermometer. The EMI shielding performances including SE_R_, SE_A_ and SE_T_ were analyzed using a PNA-N5244A vector network analyzer (Agilent).

## Results and Discussion

### Design Principle and Preparation of Segregated Nanocomposite Foams

**Fig. 1:**
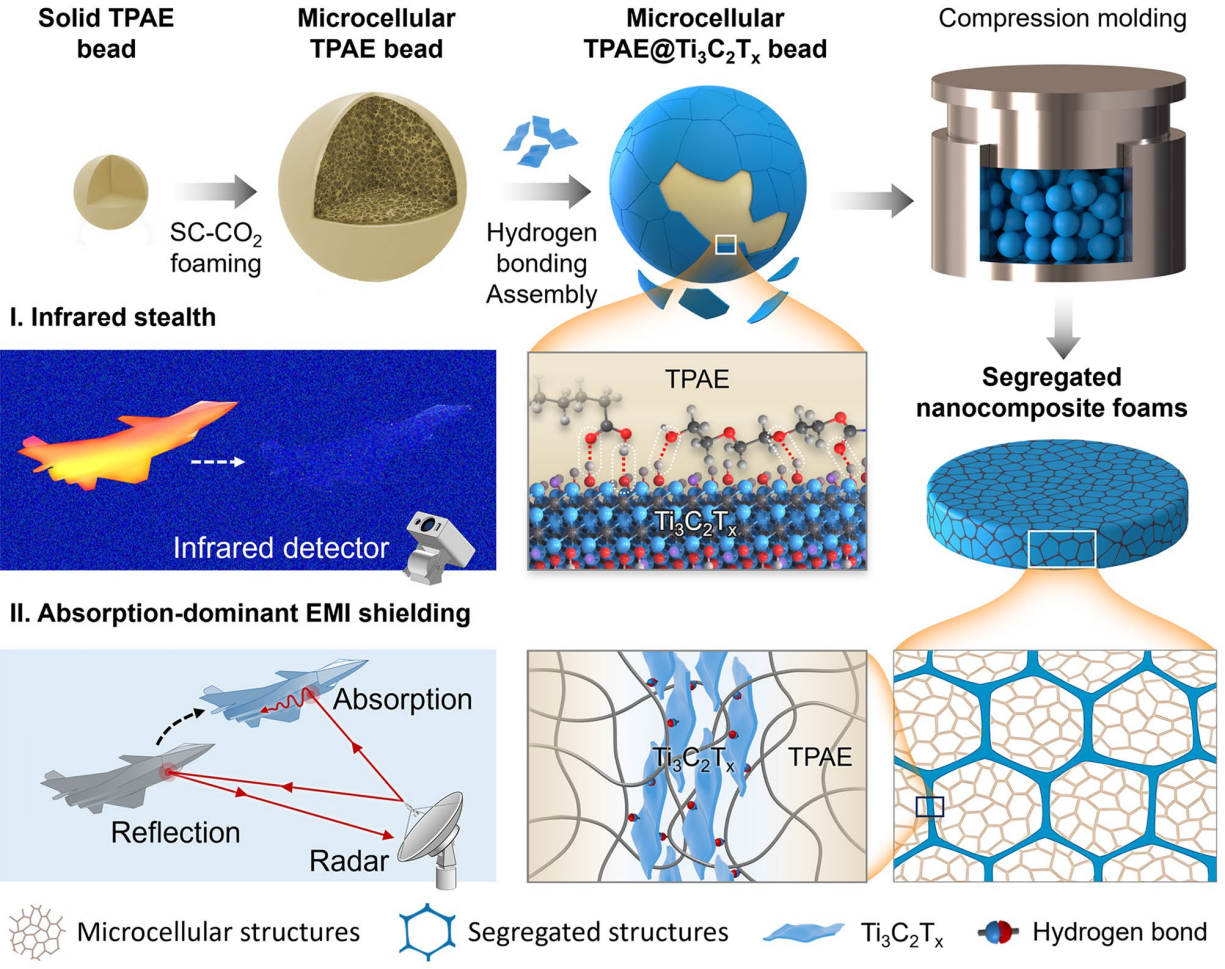
Schematic illustration for fabrication of lightweight and high-efficiency dual-functional segregated nanocomposite foams for integrated infrared stealth and absorption-dominant EMI shielding

By synchronous construction and optimization of microcellular structures and segregated structures, the lightweight and high-efficiency dual-functional segregated nanocomposite foams with integrated infrared stealth and absorption-dominant EMI shielding capacities are developed via the efficient and scalable supercritical CO_2_ (SC-CO_2_) foaming combined with hydrogen bonding assembly and compression molding strategy (Fig. 1). Briefly stated, the highly resilient microcellular TPAE beads with thin solid skins and microcellular cores are prepared by the solid-state SC-CO_2_ foaming. Subsequently, the conductive Ti_3_C_2_T_*x*_ MXene is uniformly assembled on the surfaces of microcellular TPAE beads based on the abundant hydrogen bonding interaction between the carbonyl group (C=O) in TPAE molecule chains and hydroxyl group (–OH) on Ti_3_C_2_T_*x*_ MXene. After compression molding, the lightweight and high-efficiency dual-functional segregated nanocomposite foams are obtained. The resultant segregated nanocomposite foams exhibit excellent interface adhesion and dynamic mechanical properties owing to the physical entanglement and hydrogen bonding interactions and show superior infrared stealth and absorption-dominant EMI shielding performances. Firstly, the synergistic effect of highly effective thermal insulation and low infrared emissivity endows the segregated nanocomposite foams with superior infrared stealth performances upon the infrared object. Secondly, the excellent absorption-dominant EMI shielding performances are achieved owing to the synchronous construction of microcellular structures and segregated structures. Moreover, the segregated nanocomposite foams exhibit outstanding working reliability and stability upon dynamic compression cycles. Therefore, the resultant segregated nanocomposite foams are expected to be used as lightweight and high-efficiency dual-functional infrared stealth and absorption-dominant EMI shielding materials in aerospace, weapons, military and wearable electronics.

### Morphologies of Microcellular TPAE Beads and Ti_3_C_2_T_*x*_ MXene

**Fig. 2:**
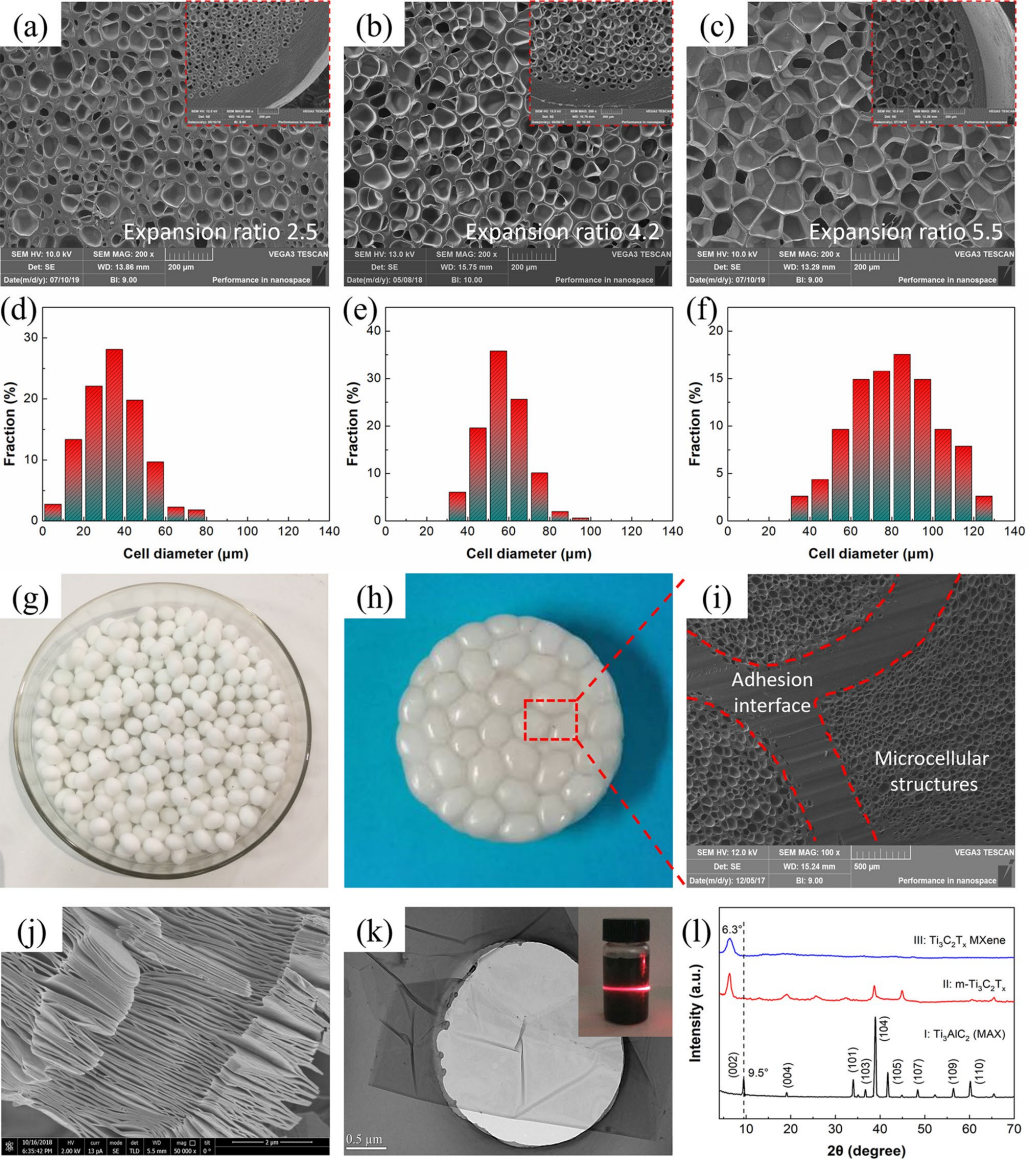
**a–c** SEM images and **d–f** cell-size distributions of the microcellular TPAE beads with different expansion ratios. **g** Digital images of the microcellular TPAE beads. **h** Digital and **i** SEM images of the microcellular TPAE foams. **j** SEM image of the m-Ti_3_C_2_T_*x*_. **k** TEM image of the Ti_3_C_2_T_x_ MXene. **l** XRD patterns of the Ti_3_AlC_2_, m-Ti_3_C_2_T_*x*_ and Ti_3_C_2_T_*x*_ MXene

Figure 2a-c shows the cellular morphologies of microcellular TPAE beads with expansion ratios of 2.5, 4.2 and 5.5 (corresponding mass densities of 0.40, 0.24 and 0.18) foamed for 25, 50 and 75 s, respectively. After microcellular foaming, the TPAE beads turn from semitransparent and stiff to white opaque and highly resilient (Figs. S4 and S5). It is observed that the microcellular TPAE beads all present skin–core morphologies with uniform foamed cores and thin unfoamed skins (inset in Fig. 2a–c). With increasing foaming time, the microcellular TPAE beads exhibit thinner unfoamed skins and more highly foamed cores with larger cell size, smaller cell density and cell wall thickness. This is because that the dissolved CO_2_ molecules in TPAE matrix continuously diffuse into the initially nucleated cells, resulting in the larger cell size, smaller cell density and thinner cell wall due to the uniaxial compression and biaxial tension effects during microcellular foaming. The statistically calculated cell diameters of microcellular TPAE beads foamed for 25, 50 and 75 s are 39.5, 65.8 and 93.2 μm with large cell densities of 4.93 × 10^6^, 2.65 × 10^6^ and 1.07 × 10^6^ cells cm^−3^, and cell wall thicknesses of 16.8, 10.2 and 3.9 μm, respectively. For the formation of unfoamed solid skins, it is deduced that after saturation and pressure release, the CO_2_ molecules in the skin region begin to diffuse outward, resulting in the relatively lower gas concentration and thus decreased foamability. As shown in Fig. 2h, i, the microcellular TPAE foams with good interfacial adhesion and well-maintained microcellular structures are feasibly fabricated by compression molding of the microcellular TPAE beads, which show unique skin–core morphologies with thin unfoamed skins and highly elastic foamed cores. Specifically, the partially dissolved TPAE molecules on the surfaces of adjacent microcellular TPAE beads diffuse rapidly and tangle with each other during compression molding, forming the strong adhesion interfaces between adjacent microcellular TPAE beads (Fig. S6). Meanwhile, the microcellular TPAE beads show excellent flexibility with adaptable microcellular structures upon the compression deformation and exhibit well-maintained microcellular structures after compression molding due to their outstanding rebound resilience. Figure S7 demonstrates the successful fabrication of large-scale microcellular TPAE foams with bigger dimensions based on the microcellular TPAE beads.

Figures 2j and S8 show the SEM images of Ti_3_AlC_2_ and multilayer Ti_3_C_2_T_*x*_ (m-Ti_3_C_2_T_*x*_). After chemically etching the Al layers, the m-Ti_3_C_2_T_*x*_ shows accordion-like structures with loosely stacked Ti_3_C_2_T_*x*_ nanosheets. This facilitates the exfoliation of Ti_3_C_2_T_*x*_ MXene owing to the weakened interlayer interactions. The obtained few-layer Ti_3_C_2_T_*x*_ MXene exhibits ultrathin and highly transparent features with a large lateral size of 3.5 μm (Fig. 2k). The strong Tyndall effect of Ti_3_C_2_T_*x*_ dispersion verifies their colloidal characteristics and the high dispersibility of Ti_3_C_2_T_*x*_ MXene in DI water owing to the abundant functional groups of –O, –OH and –F. Figure 2l shows the XRD patterns of Ti_3_AlC_2_, m-Ti_3_C_2_T_*x*_ and Ti_3_C_2_T_*x*_ MXene. The disappearance of (101), (103), (104) and (105) characteristic peaks and left shift of (002) peak from 9.5° to 6.4° demonstrate the successful synthesis of Ti_3_C_2_T_*x*_ MXene with enlarged interlayer spacing. Importantly, the existence of abundant functional groups is beneficial to the hydrogen bonding assembly of Ti_3_C_2_T_*x*_ MXene on the surfaces of microcellular TPAE beads by convenient dip-coating process.

### Morphologies of Segregated Nanocomposite Foams

**Fig. 3:**
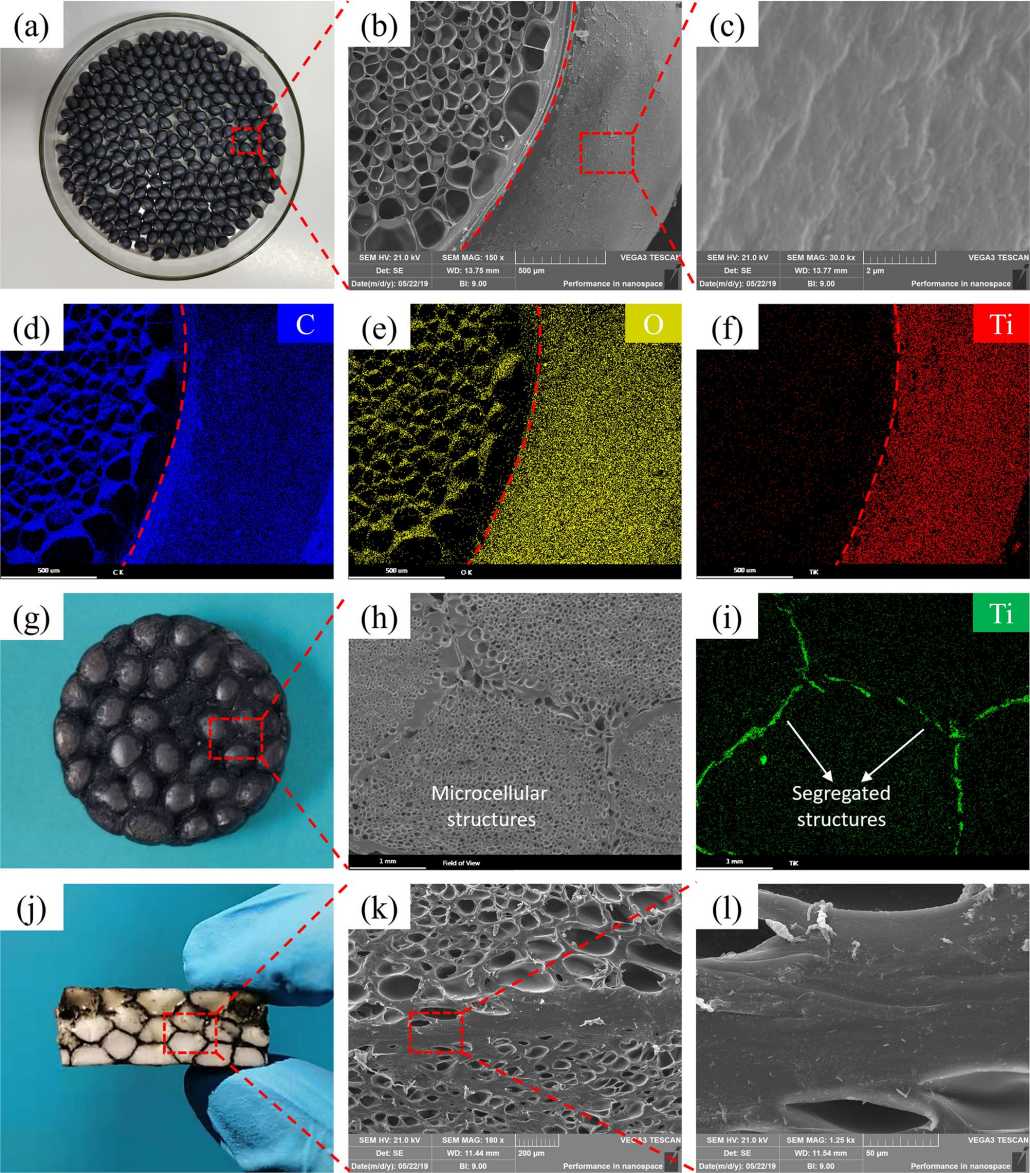
**a** Digital and **b, c** SEM images of the microcellular TPAE@Ti_3_C_2_T_*x*_ beads. **d**–**f** EDS mapping images of C, O and Ti elements of the microcellular TPAE@Ti_3_C_2_T_*x*_ beads. **g** Digital, **h** SEM and **i** EDS mapping images of the segregated nanocomposite foams. **j** Digital image of the fracture surface of segregated nanocomposite foams. **k, l** SEM images of the interface adhesion of segregated nanocomposite foams

Figure 3a–c shows the surface and interior morphologies of microcellular TPAE@Ti_3_C_2_T_*x*_ beads with the expansion ratio of 4.2. As can be seen, the Ti_3_C_2_T_*x*_ MXene is uniformly assembled on the surfaces of microcellular TPAE beads with a black surface, thanks to the abundant hydrogen bonding interaction between the carbonyl group (C=O) in TPAE molecule chains and hydroxyl group (–OH) on the surface of Ti_3_C_2_T_*x*_ MXene. The corresponding EDS mappings of C, O and Ti elements also demonstrate the uniform assembly of Ti_3_C_2_T_*x*_ MXene on the surfaces of microcellular TPAE beads (Fig. 3d–f). Figure 3g–i shows the digital, SEM and EDS mapping images of the segregated nanocomposite foams with an expansion ratio of 4.2. They evidently demonstrate the synchronous construction of microcellular structures and segregated structures. The Ti_3_C_2_T_*x*_ MXene is selectively distributed at the interfaces of adjacent microcellular TPAE beads, forming the highly efficient three-dimensional (3D) continuous conductive networks at ultralow Ti_3_C_2_T_*x*_ contents. The introduction of microcellular structures endows the segregated nanocomposite foams with lightweight and high resilience. For instance, the segregated nanocomposite foams with an expansion ratio of 5.5 exhibit a low mass density of 0.32 g cm^−3^ (Fig. S10) and can be floated on the water (Fig. S11). Figure 3j–l shows the interfacial morphologies of the segregated nanocomposite foams. The segregated nanocomposite foams present good interfacial adhesion with orientationally aligned Ti_3_C_2_T_*x*_ MXene at the adhesion interfaces, which is beneficial to obtain the highly efficient 3D continuous conductive networks at ultralow Ti_3_C_2_T_*x*_ content. The strong adhesion interfaces of the segregated nanocomposite foams mainly benefit from two reasons. On the one hand, the molecular chains on the surfaces of adjacent microcellular TPAE beads diffuse and entangle with each other during compression molding, leading to the physical anchoring of Ti_3_C_2_T_*x*_ MXene. On the other hand, the hydrogen bonding interaction between C=O in TPAE molecule chains and   –OH on the surface of Ti_3_C_2_T_*x*_ MXene strengthens the adhesion interfaces of segregated nanocomposite foams.

**Fig. 4:**
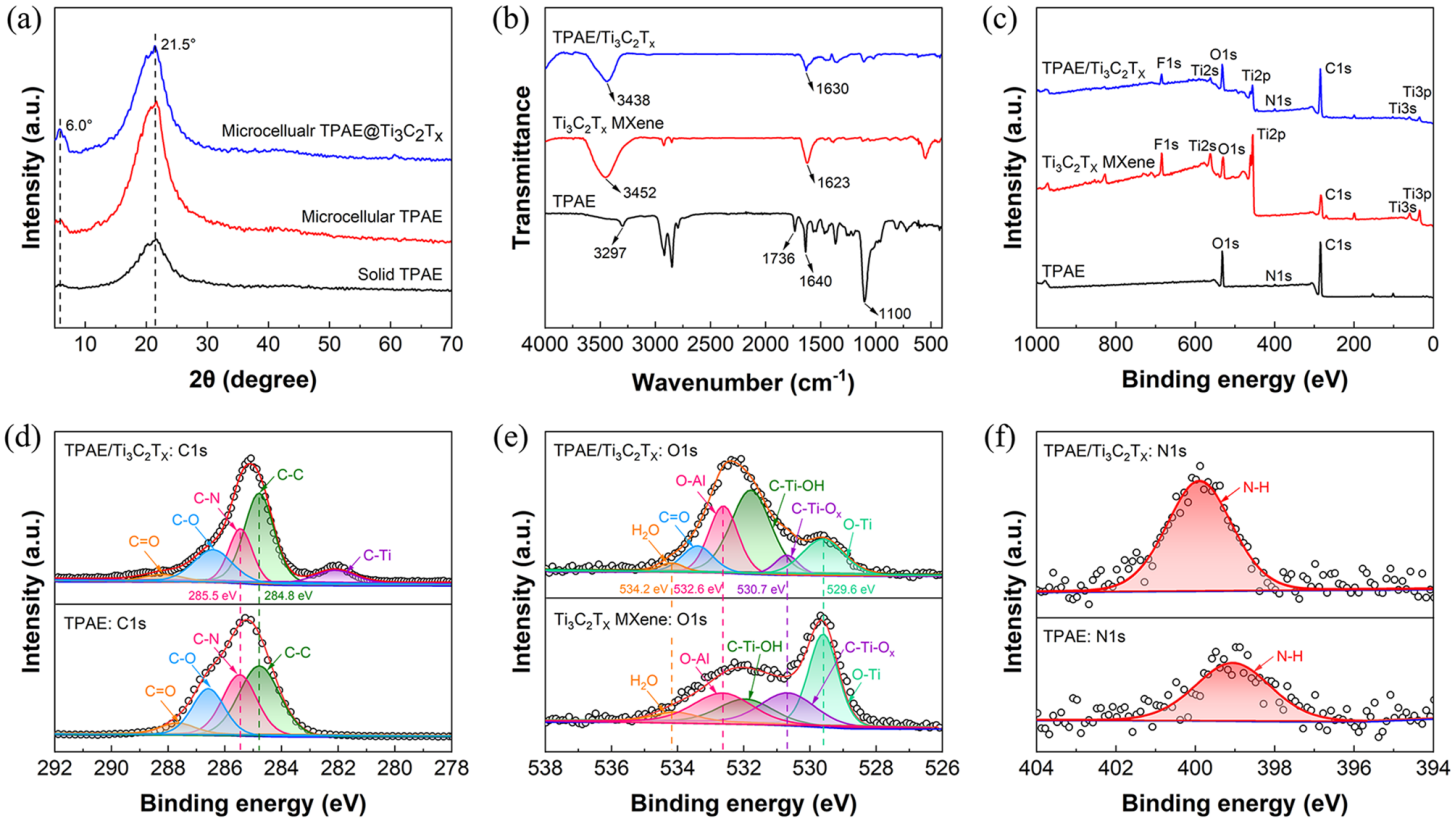
**a** XRD patterns of the solid and microcellular TPAE beads, as well as microcellular TPAE@Ti_3_C_2_T_*x*_ beads. **b** FTIR and **c** XPS spectra of the TPAE, Ti_3_C_2_T_*x*_ MXene and TPAE/Ti_3_C_2_T_*x*_ nanocomposites. High-resolution XPS spectra of **d** C 1*s* for TPAE and TPAE/Ti_3_C_2_T_*x*_ nanocomposites, **e** O 1*s* for Ti_3_C_2_T_*x*_ MXene and TPAE/Ti_3_C_2_T_*x*_ nanocomposites, and **f** N 1*s* for TPAE and TPAE/Ti_3_C_2_T_*x*_ nanocomposites

The chemical structures and hydrogen bonding interactions between TPAE and Ti_3_C_2_T_*x*_ MXene were investigated by XRD, FTIR and XPS. As shown in Fig. 4a, the microcellular TPAE beads exhibit an enhanced intensity of *γ*-form crystals at 21.5° compared with the solid beads, owing to the plasticization and rearrangement of molecular chains during SC-CO_2_ foaming. After assembly of Ti_3_C_2_T_*x*_ MXene on the surface of microcellular TPAE beads, the diffraction peak at 21.5° weakens, and the diffraction peak corresponding to (002) of Ti_3_C_2_T_*x*_ MXene appears at 6.0°. Figure 4b shows the FTIR spectra of TPAE, Ti_3_C_2_T_*x*_ MXene and TPAE/Ti_3_C_2_T_*x*_ nanocomposites. Compared with the pure TPAE and Ti_3_C_2_T_*x*_ MXene, the C=O characteristic peak of TPAE/Ti_3_C_2_T_x_ nanocomposites is shifted from 1640 to 1630 cm^−1^, and the  –OH characteristic peak is shifted from 3452 to 3438 cm^−1^. Therefore, the chemical environment of C =O and  –OH has been changed, indicating the formation of hydrogen bonding interactions between TPAE and Ti_3_C_2_T_*x*_ MXene with C=O as proton acceptor and  –OH as proton donor. Figure 4c–f shows the XPS wide-scan spectra and high-resolution spectra of TPAE, Ti_3_C_2_T_*x*_ MXene and TPAE/Ti_3_C_2_T_*x*_ nanocomposites. As can be seen, the TPAE/Ti_3_C_2_T_*x*_ nanocomposites show distinct Ti and F characteristic peaks due to the introduction of Ti_3_C_2_T_*x*_ MXene. For the TPAE/Ti_3_C_2_T_*x*_ nanocomposites, the C=O characteristic peak of TPAE shifts from 287.7 to 288.2 eV in the C 1*s* spectra (Fig. 4d), the C–Ti–OH characteristic peak of Ti_3_C_2_T_*x*_ MXene shifts from 531.9 to 531.8 eV in the O 1*s* spectra (Fig. 4e), and the N–H characteristic peak of TPAE shifts from 399.1 to 399.9 eV in the N 1*s* spectra (Fig. 4f). This indicates that the chemical environments of C=O and N–H in TPAE and C–Ti–OH in Ti_3_C_2_T_*x*_ MXene have been changed, demonstrating the formation of hydrogen bonding interactions between TPAE and Ti_3_C_2_T_*x*_ MXene. The synergetic effect of physical entanglement and hydrogen bonding interactions contributes to the enhanced adhesion interfaces and improved mechanical properties of segregated nanocomposite foams.

### Infrared Stealth Performances of Segregated Nanocomposite Foams

**Fig. 5:**
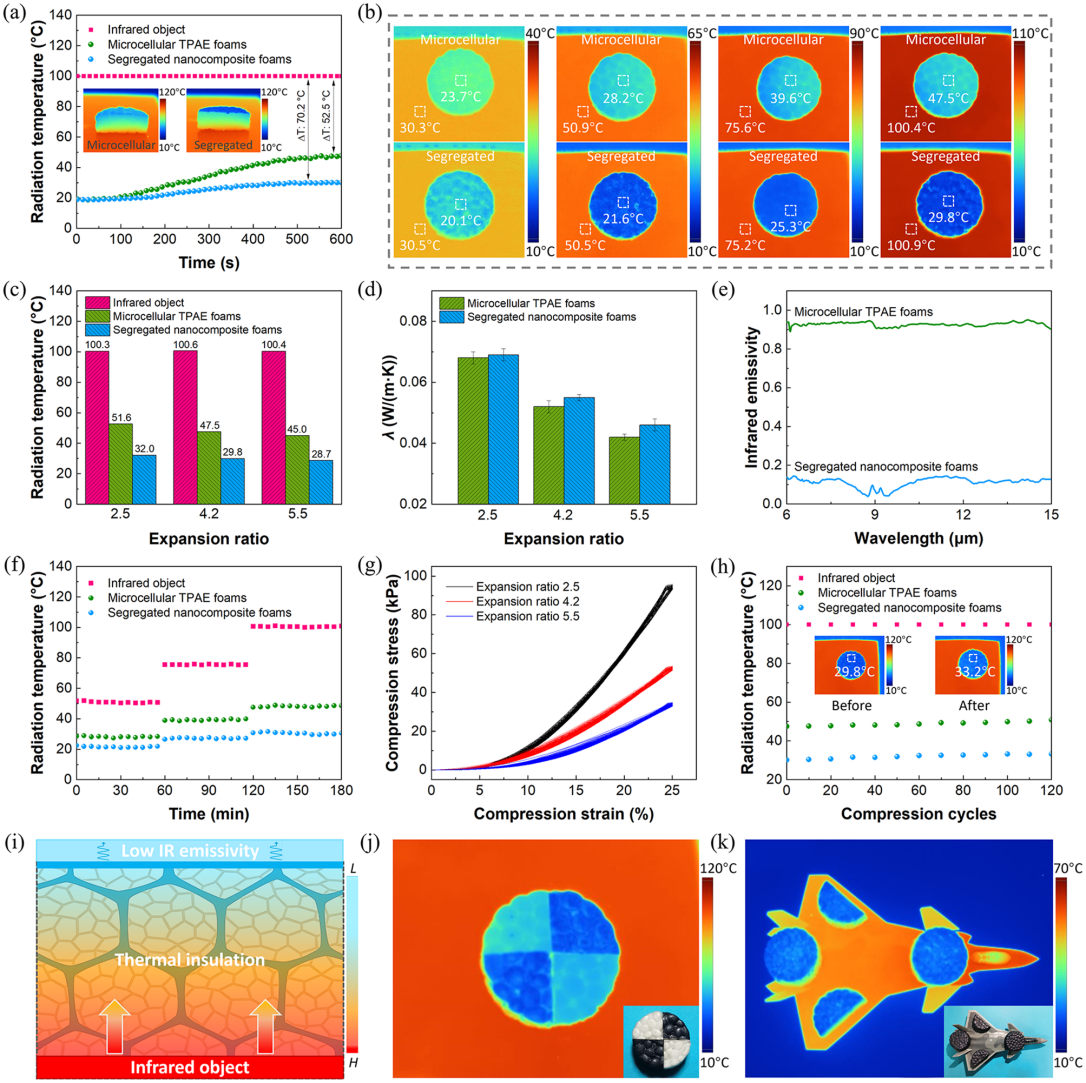
**a** Radiation temperatures of the microcellular TPAE foams and segregated nanocomposite foams with an infrared object temperature of 100 °C. **b** Infrared images of the microcellular TPAE foams and segregated nanocomposite foams at different object temperatures of 30, 50, 75 and 100 °C. **c** Radiation temperatures of the microcellular TPAE foams and segregated nanocomposite foams with different expansion ratios. **d** Thermal conductivities and **e** infrared emissivity of the microcellular TPAE foams and segregated nanocomposite foams. **f** Long-term infrared stealth performances of the microcellular TPAE foams and segregated nanocomposite foams. **g** Cycling compression behaviors of the segregated nanocomposite foams with different expansion ratios for 120 circles. **h** Infrared stealth stabilities of the microcellular TPAE foams and segregated nanocomposite foams upon repeated compression strains. **i** Infrared stealth mechanisms of the segregated nanocomposite foams. **j** Infrared image of the diagonally recombined microcellular TPAE foams and segregated nanocomposite foams. **k** Infrared stealth of the airplane model covered by segregated nanocomposite foams

The infrared stealth performances of microcellular TPAE foams and segregated nanocomposite foams with the same thickness of 8 mm are evaluated on the hot stage simulating the infrared object at various temperatures. The Ti_3_C_2_T_*x*_ dispersion concentration used for dip-coating is 20 mg mL^−1^. Figure 5a shows the radiation temperatures of microcellular TPAE foams and segregated nanocomposite foams (expansion ratio of 4.2) with a consistent object temperature of 100 °C. As can be seen, the radiation temperatures of microcellular TPAE foams and segregated nanocomposite foams gradually rise to the low steady values of 47.5 and 29.8 °C with the *∆T* of 52.5 and 70.2 °C, respectively, compared with the object temperature, indicating the infrared stealth capacities of microcellular TPAE foams and segregated nanocomposite foams. Notably, the segregated nanocomposite foams exhibit much better infrared stealth performances with a larger *∆T* than the microcellular TPAE foams. From the infrared images in Fig. 5a, it is also observed that the upper surface of segregated nanocomposite foams possesses a lower radiation temperature than that of microcellular TPAE foams. Figure 5b shows that at the different object temperatures of 30, 50, 75 and 100 °C, the segregated nanocomposite foams all present much lower radiation temperatures compared with the microcellular TPAE foams, indicating their superior infrared stealth performances.

Figures 5c and S12 show that the segregated nanocomposite foams with expansion ratios of 2.5, 4.2 and 5.5 all exhibit better infrared stealth performances compared with the microcellular TPAE foams. With the increasing expansion ratio, the radiation temperatures of both microcellular TPAE foams and segregated nanocomposite foams decrease slightly. According to Stefan–Boltzmann law: *E* = *εσT*^4^, where *σ* refers to the Stefan–Boltzmann constant, the thermal radiation energy is directly dependent on the surface infrared emissivity (*ε*) and surface absolute temperature (*T*) [65]. As shown in Fig. 5d, the microcellular TPAE foams and segregated nanocomposite foams exhibit approximately the similar low thermal conductivities (*λ*) benefitting from the incorporation of microcellular structures, indicating their outstanding thermal insulating features. For instance, the microcellular TPAE foams and segregated nanocomposite foams with an expansion ratio of 4.2 exhibit low *λ* values of 0.052 and 0.055 W m^−1^ K^−1^, respectively. With the increasing expansion ratio, the *λ* values of them both decrease gradually. Figure 5e shows that the segregated nanocomposite foams possess an ultralow average infrared emissivity of 0.13 compared with the microcellular TPAE foams (0.88), which may benefit from the low infrared emissivity of Ti_3_C_2_T_*x*_ MXene [66, 67]. Therefore, compared with the microcellular TPAE foams with only thermal insulation dominated infrared stealth, the segregated nanocomposite foams exhibit superior infrared stealth performances owing to the synergistic effect of highly effective thermal insulation of microcellular structures and low infrared emissivity of Ti_3_C_2_T_*x*_ MXene (Fig. S13).

Figure 5f shows the long-term infrared stealth performances of microcellular TPAE foams and segregated nanocomposite foams at the object temperatures of about 50, 75 and 100 °C, respectively. As can be seen, the microcellular TPAE foams and segregated nanocomposite foams both present steady surface radiation temperatures during the duration of 3 h at different object temperatures, demonstrating their excellent working stability and reliability in infrared stealth. Figures 5g and S15 show the cycling compression behaviors of segregated nanocomposite foams with different expansion ratios for 120 loading–unloading circles with a maximum strain of 25%. Thanks to the intrinsic high resilience of TPAE and incorporation of microcellular structures, the segregated nanocomposite foams exhibit excellent cyclic mechanical stability during dynamic loadings with nearly coincident stress–strain curves and negligible hysteresis rings. With larger expansion ratio, the segregated nanocomposite foams exhibit improved flexibility with lower compression stress and compression modulus. The outstanding tensile properties of segregated nanocomposite foams (expansion ratio: 4.2) with a high tensile strength of 2.05 MPa and a large tensile strain at break of 296.3% also demonstrate the excellent interfacial adhesion between microcellular TPAE@Ti_3_C_2_T_*x*_ beads (Fig. S16). The infrared stealth performances of microcellular TPAE foams and segregated nanocomposite foams upon repeated compression strains are evaluated, as shown in Fig. 5h. Note that the segregated nanocomposite foams exhibit superior and steady infrared stealth performances with the radiation temperature maintained at low values even after 120 repeated compression cycles, demonstrating their excellent infrared stealth reliability and stability upon mechanical deformations. Figure 5i illustrates the infrared stealth mechanisms of segregated nanocomposite foams. Benefitting from the incorporation of microcellular structures, the segregated nanocomposite foams covered on the high-temperature infrared object exhibit lower surface temperature owing to their highly effective thermal insulation, which is similar to the microcellular TPAE foams. Meanwhile, the low infrared emissivity of segregated nanocomposite foams with assembled Ti_3_C_2_T_*x*_ MXene further dramatically decreases the surface radiation temperatures. Therefore, the segregated nanocomposite foams exhibit superior infrared stealth performances owing to the synergistic effect of highly effective thermal insulation and low infrared emissivity. Figure 5j shows the infrared image of diagonally recombined sample by two quarters of microcellular TPAE foams and two quarters of segregated nanocomposite foams. The locally distributed radiation temperatures prove the superior infrared stealth capacities of segregated nanocomposite foams. Figure 5k shows that the airplane model covered by segregated nanocomposite foams can realize selectively concealing under the thermal imager, demonstrating their promising application potentials in aerospace infrared stealth.

### EMI Shielding Performances of Segregated Nanocomposite Foams

**Fig. 6:**
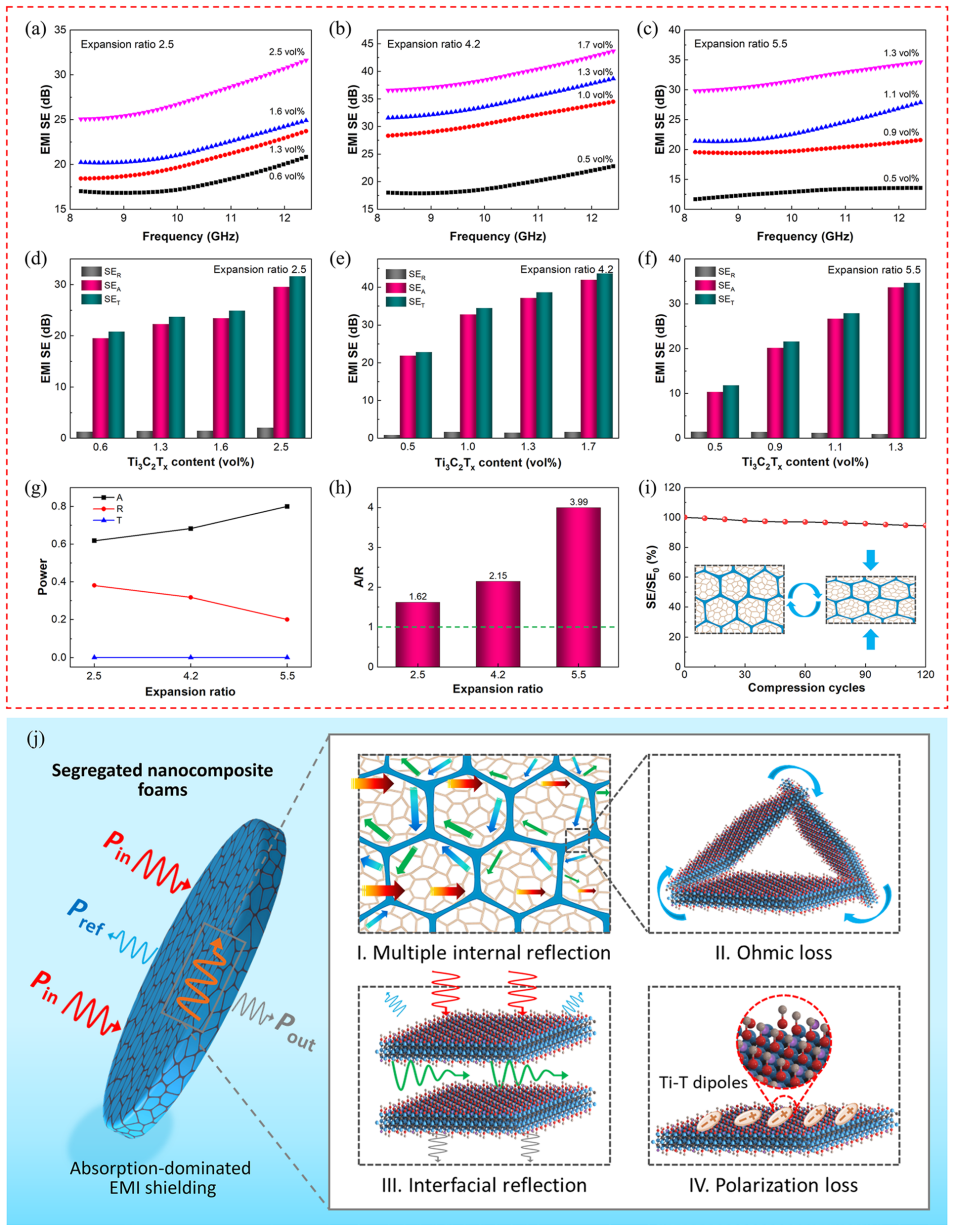
**a–c** Total EMI SE of the segregated nanocomposite foams with different expansion ratios and Ti_3_C_2_T_*x*_ contents. **d–f** SE_R_, SE_A_ and SE_T_ of the segregated nanocomposite foams with different expansion ratios and Ti_3_C_2_T_*x*_ contents. **g** *A*, *R* and *T* coefficients of the segregated nanocomposite foams with different expansion ratios. **h** *A*/*R* of the segregated nanocomposite foams with different expansion ratios. **i** Relative SE of the segregated nanocomposite foams upon repeated compression. **j** EMI shielding mechanism of the segregated nanocomposite foams

Figure 6a–c shows the EMI shielding performances of segregated nanocomposite foams with different microcellular TPAE bead expansion ratios and Ti_3_C_2_T_*x*_ contents. The segregated nanocomposite foams with tailorable Ti_3_C_2_T_*x*_ contents are obtained by simply changing the Ti_3_C_2_T_*x*_ dispersion concentration during dip-coating. With the increasing Ti_3_C_2_T_*x*_ content, the segregated nanocomposite foams with different expansion ratios (thickness: 8 mm) all exhibit significantly improved EMI SE owing to the more efficient 3D conductive networks and higher electrical conductivity (Fig. S17). The EDS mapping images in Fig. S18 also indicate the formation of continuous segregated conductive networks at ultralow Ti_3_C_2_T_*x*_ contents. The segregated nanocomposite foams with an expansion ratio of 2.5, for instance, exhibit a total EMI SE of 32 dB at the low Ti_3_C_2_T_*x*_ content of 2.5 vol%, which is sufficient for the commercial application requirements (> 20 dB). When the microcellular TPAE bead expansion ratio is increased to 4.2, the segregated nanocomposite foams with a lower Ti_3_C_2_T_*x*_ content of 1.7 vol% exhibit an enhanced total EMI SE of 44 dB to meet the higher demand of high-tech applications although the electrical conductivity is decreased. Figure 6d, e shows the corresponding microwave refection (SE_R_), microwave absorption (SE_A_) and total EMI SE (SE_T_) of segregated nanocomposite foams with expansion ratios of 2.5 and 4.2, respectively. Note that the segregated nanocomposite foams with the larger expansion ratio of 4.2 and lower Ti_3_C_2_T_*x*_ content of 1.7 vol% exhibit significantly increased SE_T_ (44 dB) and SE_A_ (42 dB) with a decreased SE_R_ (2 dB) than those with an expansion ratio of 2.5 and a Ti_3_C_2_T_*x*_ content of 2.5 vol%. It is because that the introduction of more microcellular structures in segregated nanocomposite foams with larger millimeter-scale segregated conductive networks can improve the impedance matching due to the decreased electrical conductivity, thus allowing more penetration of incident EM waves in the segregated nanocomposite foams with less direct reflection on the surfaces. This consequently induces more multiple internal reflection and scattering of EM waves within the millimeter-scale segregated conductive networks, resulting in the enhanced attenuation of EM waves via absorption and thus absorption-dominant EMI shielding. With the higher expansion ratio of 5.5, nevertheless, the segregated nanocomposite foams exhibit a slightly decreased EMI SE of 35 dB (Fig. 6c, f), which could result from the decreased absorption loss of EM waves within the less segregated conductive networks at the same thickness. Interestingly, all the segregated nanocomposite foams exhibit increased total EMI SE upon the increasing EM wave frequency, indicating that the high‑frequency EM waves attenuate more efficiently within the millimeter-scale segregated conductive networks owing to their shorter wavelength with closer trough–crest distance.

The absorptivity (*A*), reflectivity (*R*) and transmissivity (*T*) coefficients are calculated by the scattering parameters to evaluate the EMI shielding mechanisms of segregated nanocomposite foams. As shown in Figs. 6g and S19, the segregated nanocomposite foams with three expansion ratios of 2.5, 4.2 and 5.5 all exhibit high *A* values above 0.6 and low *R* values below 0.4. The larger expansion ratio results in the higher *A* value and lower *R* value. The lower *R* value and the larger *A* value indicate the more EM power attenuated by absorption within the hierarchical cellular structures [68–70]. The segregated nanocomposite foams with the expansion ratio of 4.2 possess a low *R* value below 0.3, and those with a larger expansion ratio of 5.5 possess an even lower *R* value around 0.2. Correspondingly, the segregated nanocomposite foams with three expansion ratios exhibit high *A*/*R* ratios of 1.62, 2.15 and 3.99, respectively, which are much larger than 1.0 (Fig. 6h). This demonstrates that the segregated nanocomposite foams exhibit absorption-dominant EMI shielding behaviors with most of the incident EM waves attenuated through absorption instead of reflection, which is beneficial to reduce the secondary pollution of EM waves. Figure 6i shows the relative SE (SE/SE_0_ × 100%) of segregated nanocomposite foams upon the repeated compression. After the 120 repeated compression cycles with a compression strain of 25%, the relative SE still presents a high retention rate above 94.5%, demonstrating the outstanding EMI shielding stability of segregated nanocomposite foams after dynamic mechanical deformations.

The absorption-dominant EMI shielding mechanisms of segregated nanocomposite foams mainly benefit from the synchronous construction of microcellular structures and segregated structures. Figure 6j schematically illustrates the propagation of EM waves across the segregated nanocomposite foams. Thanks to the incorporation of microcellular structures, most of the incident EM waves enter the segregated nanocomposite foams with low direct reflection owing to the improved surface impedance matching. In the segregated structures containing numerous microcellular structures, the EM waves will be attenuated via multiple internal reflection and scattering on the interfaces. Meanwhile, the EM waves can be attenuated by interacting with the electron carriers in the conductive networks, leading to the ohmic losses of EM waves. Moreover, the multiple interfacial reflections occurred between the neighboring Ti_3_C_2_T_*x*_ nanosheets also contribute to the dissipation of EM waves. In addition, the localized imperfections and terminal groups including –O–, –F and –OH on the surfaces of Ti_3_C_2_T_*x*_ MXene induce the uneven distribution of charge density, causing the creation of local dipoles upon the EM field and increased polarization loss. The unique hierarchical segregated microcellular structures act as the role of “black hole”, which can efficiently absorb the EM waves and prevent them from escaping. Therefore, the obtained segregated nanocomposite foams exhibit superior absorption-dominant EMI shielding performances. Figure S20 demonstrates that the segregated nanocomposite foams possess certain long-term infrared stealth and EMI shielding working stabilities in the air environment. The results demonstrate that the lightweight and high-efficiency dual-functional segregated nanocomposite foams with integrated infrared stealth and absorption-dominant EMI shielding capacities possess excellent potentials in areas of aerospace, weapons, military and wearable electronics.

## Conclusions

In summary, this work demonstrates the development of lightweight and high-efficiency dual-functional segregated nanocomposite foams for infrared stealth and absorption-dominant EMI shielding via the efficient and scalable supercritical CO_2_ foaming combined with hydrogen bonding assembly and compression molding strategy. The chemical structures, hierarchical morphologies, electrical and mechanical properties as well as infrared stealth and EMI shielding performances as functions of microcellular TPAE bead expansion ratio and Ti_3_C_2_T_*x*_ content are investigated in detail. Benefitting from the synchronous construction of microcellular structures and segregated structures, the nanocomposite foams exhibit lightweight, improved flexibility and resilience, as well as desirable electrical conductivities at the ultralow Ti_3_C_2_T_*x*_ contents. The synergetic effect of physical entanglement and hydrogen bonding interactions between TPAE and Ti_3_C_2_T_*x*_ MXene results in the excellent adhesion interfaces and dynamic mechanical properties. The resultant segregated nanocomposite foams show superior infrared stealth performances (with a large radiation temperature reduction of 70.2 °C at the object temperature of 100 °C) thanks to the synergistic effect of highly effective thermal insulation and low infrared emissivity, and excellent absorption-dominant EMI shielding performances (with a high *A*/*R* ratio of 2.15) owing to the multiple internal reflections within segregated structures, massive ohmic loss, interfacial reflection and polarization loss of EM waves. Moreover, the segregated nanocomposite foams exhibit outstanding infrared stealth and EMI shielding stability upon dynamic compression cycles. We believe that the lightweight and high-efficiency dual-functional segregated nanocomposite foams with integrated infrared stealth and absorption-dominant EMI shielding capacities have promising potentials for applications in aerospace, weapons, military and wearable electronics.

## Supplementary Information

Below is the link to the electronic supplementary material.

Supplementary file1 (PDF 1256 KB)

## References

[1] A. Iqbal, F. Shahzad, K. Hantanasirisakul, M.K. Kim, J. Kwon et al., Anomalous absorption of electromagnetic waves by 2D transition metal carbonitride Ti_3_CNT_x_ (MXene). Science **369**, 446–450 (2020). 10.1126/science.aba7977 32703878 10.1126/science.aba7977

[2] Y. Wu, S. Tan, Y. Zhao, L. Liang, M. Zhou et al., Broadband multispectral compatible absorbers for radar, infrared and visible stealth application. Prog. Mater. Sci. **135**, 101088 (2023). 10.1016/j.pmatsci.2023.101088

[3] M. Wu, Z. Shao, N. Zhao, R. Zhang, G. Yuan et al., Knittable aerogel fiber for thermal insulation textile. Science **382**, 1379–1383 (2023). 10.1126/science.adj8013 38127754 10.1126/science.adj8013

[4] Z. Ma, X. Xiang, L. Shao, Y. Zhang, J. Gu Multifunctional wearable silver nanowire decorated leather nanocomposites for Joule heating, electromagnetic interference shielding and piezoresistive sensing. Angew. Chem. Int. Ed. **61**, e202200705 (2022). 10.1002/anie.202200705 10.1002/anie.202200705 35122674

[5] Y. Sun, X. Han, P. Guo, Z. Chai, J. Yue et al., Slippery graphene-bridging liquid metal layered heterostructure nanocomposite for stable high-performance electromagnetic interference shielding. ACS Nano **17**, 12616–12628 (2023). 10.1021/acsnano.3c02975 37382511 10.1021/acsnano.3c02975

[6] X. Ma, J. Pan, H. Guo, J. Wang, C. Zhang et al., Ultrathin wood-derived conductive carbon composite film for electromagnetic shielding and electric heating management. Adv. Funct. Mater. **33**, 2213431 (2023). 10.1002/adfm.202213431

[7] B.-F. Guo, Y.-J. Wang, C.-F. Cao, Z.-H. Qu, J. Song et al., Large-scale, mechanically robust, solvent-resistant, and antioxidant MXene-based composites for reliable long-term infrared stealth. Adv. Sci. **11**, e2309392 (2024). 10.1002/advs.202309392 10.1002/advs.202309392 PMC11077694 38403451

[8] Y.-Y. Shi, S.-Y. Liao, Q.-F. Wang, X.-Y. Xu, X.-Y. Wang et al., Enhancing the interaction of carbon nanotubes by metal-organic decomposition with improved mechanical strength and ultra-broadband EMI shielding performance. Nano-Micro Lett. **16**, 134 (2024). 10.1007/s40820-024-01344-1 10.1007/s40820-024-01344-1 PMC10899147 38411757

[9] Z. Zhuang, H. Chen, C. Li, Robust pristine MXene films with superhigh electromagnetic interference shielding effectiveness via spatially confined evaporation. ACS Nano **17**, 10628–10636 (2023). 10.1021/acsnano.3c01697 37259949 10.1021/acsnano.3c01697

[10] B.-X. Li, Z. Luo, W.-G. Yang, H. Sun, Y. Ding et al., Adaptive and adjustable MXene/reduced graphene oxide hybrid aerogel composites integrated with phase-change material and thermochromic coating for synchronous visible/infrared camouflages. ACS Nano **17**, 6875–6885 (2023). 10.1021/acsnano.3c00573 36996266 10.1021/acsnano.3c00573

[11] Y.-Y. Wang, F. Zhang, N. Li, J.-F. Shi, L.-C. Jia et al., Carbon-based aerogels and foams for electromagnetic interference shielding: a review. Carbon **205**, 10–26 (2023). 10.1016/j.carbon.2023.01.007

[12] Z. Deng, L. Li, P. Tang, C. Jiao, Z.Z. Yu et al., Controllable surface-grafted MXene inks for electromagnetic wave modulation and infrared anti-counterfeiting applications. ACS Nano **16**, 16976–16986 (2022). 10.1021/acsnano.2c07084 36197991 10.1021/acsnano.2c07084

[13] Z. Zeng, F. Jiang, Y. Yue, D. Han, L. Lin et al., Flexible and ultrathin waterproof cellular membranes based on high-conjunction metal-wrapped polymer nanofibers for electromagnetic interference shielding. Adv. Mater. **32**, e1908496 (2020). 10.1002/adma.201908496 32227390 10.1002/adma.201908496

[14] M. Huang, L. Wang, X. Li, Z. Wu, B. Zhao et al., Magnetic interacted interaction effect in MXene skeleton: enhanced thermal-generation for electromagnetic interference shielding. Small **18**, e2201587 (2022). 10.1002/smll.202201587 35676238 10.1002/smll.202201587

[15] P. Yi, H. Zou, Y. Yu, X. Li, Z. Li et al., MXene-reinforced liquid metal/polymer fibers via interface engineering for wearable multifunctional textiles. ACS Nano **16**, 14490–14502 (2022). 10.1021/acsnano.2c04863 36094895 10.1021/acsnano.2c04863

[16] Y. Bai, B. Zhang, G. Fei, Z. Ma, Composite polymeric film for stretchable, self-healing, recyclable EMI shielding and Joule heating. Chem. Eng. J. **478**, 147382 (2023). 10.1016/j.cej.2023.147382

[17] J. Wang, Q. Li, K. Li, X. Sun, Y. Wang et al., Ultra-high electrical conductivity in filler-free polymeric hydrogels toward thermoelectrics and electromagnetic interference shielding. Adv. Mater. **34**, e2109904 (2022). 10.1002/adma.202109904 35064696 10.1002/adma.202109904

[18] J. Xie, G. Zhou, Y. Sun, F. Zhang, F. Kang et al., Multifunctional liquid metal-bridged graphite nanoplatelets/aramid nanofiber film for thermal management. Small **20**, e2305163 (2024). 10.1002/smll.202305163 38048535 10.1002/smll.202305163

[19] Z. Ma, S. Kang, J. Ma, L. Shao, Y. Zhang et al., Ultraflexible and mechanically strong double-layered aramid nanofiber-Ti_3_C_2_T_*x*_ MXene/silver nanowire nanocomposite papers for high-performance electromagnetic interference shielding. ACS Nano **14**, 8368–8382 (2020). 10.1021/acsnano.0c02401 32628835 10.1021/acsnano.0c02401

[20] L.-X. Liu, W. Chen, H.-B. Zhang, L. Ye, Z. Wang et al., Super-tough and environmentally stable aramid nanofiber@MXene coaxial fibers with outstanding electromagnetic interference shielding efficiency. Nano-Micro Lett. **14**, 111 (2022). 10.1007/s40820-022-00853-1 10.1007/s40820-022-00853-1 PMC9035413 35461406

[21] Z. Ma, S. Kang, J. Ma, L. Shao, A. Wei et al., High-performance and rapid-response electrical heaters based on ultraflexible, heat-resistant, and mechanically strong aramid nanofiber/Ag nanowire nanocomposite papers. ACS Nano **13**, 7578–7590 (2019). 10.1021/acsnano.9b00434 31244039 10.1021/acsnano.9b00434

[22] B. Zhou, Z. Li, Y. Li, X. Liu, J. Ma et al., Flexible hydrophobic 2D Ti_3_C_2_T_x_-based transparent conductive film with multifunctional self-cleaning, electromagnetic interference shielding and Joule heating capacities. Compos. Sci. Technol. **201**, 108531 (2021). 10.1016/j.compscitech.2020.108531

[23] Z. Ma, Y. Zhang, R. Jiang, L. Shao, J. Cao et al., Highly stretchable and room-temperature self-healing sheath-core structured composite fibers for ultrasensitive strain sensing and visual thermal management. Compos. Sci. Technol. **248**, 110460 (2024). 10.1016/j.compscitech.2024.110460

[24] X. Shen, J.-K. Kim, Graphene and MXene-based porous structures for multifunctional electromagnetic interference shielding. Nano Res. **16**, 1387–1413 (2023). 10.1007/s12274-022-4938-6

[25] M. Zhang, M.-S. Cao, J.-C. Shu, W.-Q. Cao, L. Li et al., Electromagnetic absorber converting radiation for multifunction. Mater. Sci. Eng. R. Rep. **145**, 100627 (2021). 10.1016/j.mser.2021.100627

[26] Z. Wei, Y. Cai, Y. Zhan, Y. Meng, N. Pan et al., Ultra-low loading of ultra-small Fe_3_O_4_ nanoparticles on nonmodified CNTs to improve green EMI shielding capability of rubber composites. Small **20**, e2307148 (2024). 10.1002/smll.202307148 37840441 10.1002/smll.202307148

[27] E. Zhu, K. Pang, Y. Chen, S. Liu, X. Liu et al., Ultra-stable graphene aerogels for electromagnetic interference shielding. Sci. China Mater. **66**, 1106–1113 (2023). 10.1007/s40843-022-2208-x

[28] Q. Wu, Z. Zeng, L. Xiao, From 2D graphene and MXene nanolayers to 3D biomimetic porous composite aerogels for electromagnetic interference shielding. Compos. Part A Appl. Sci. Manuf. **177**, 107939 (2024). 10.1016/j.compositesa.2023.107939

[29] X. Jia, B. Shen, L. Zhang, W. Zheng, Construction of compressible Polymer/MXene composite foams for high-performance absorption-dominated electromagnetic shielding with ultra-low reflectivity. Carbon **173**, 932–940 (2021). 10.1016/j.carbon.2020.11.036

[30] W. Chu, J. Li, J. Lin, W. Li, J. Xin et al., Honeycomb-like polyimide/Fe_3_O_4_@PPy foam for electromagnetic wave shielding with excellent absorption characteristics. Compos. Sci. Technol. **249**, 110489 (2024). 10.1016/j.compscitech.2024.110489

[31] R. Zhao, S. Kang, C. Wu, Z. Cheng, Z. Xie et al., Designable electrical/thermal coordinated dual-regulation based on liquid metal shape memory polymer foam for smart switch. Adv. Sci. **10**, e2205428 (2023). 10.1002/advs.202205428 10.1002/advs.202205428 PMC10015848 36658714

[32] M. Shi, Z. Song, J. Ni, X. Du, Y. Cao et al., Dual-mode porous polymeric films with coral-like hierarchical structure for all-day radiative cooling and heating. ACS Nano **17**, 2029–2038 (2023). 10.1021/acsnano.2c07293 36638216 10.1021/acsnano.2c07293

[33] D. Yu, Y. Liao, Y. Song, S. Wang, H. Wan et al., A super-stretchable liquid metal foamed elastomer for tunable control of electromagnetic waves and thermal transport. Adv. Sci. **7**, 2000177 (2020). 10.1002/advs.202000177 10.1002/advs.202000177 PMC7312308 32596119

[34] H. Cheng, Y. Pan, X. Wang, C. Liu, C. Shen et al., Ni flower/MXene-melamine foam derived 3D magnetic/conductive networks for ultra-efficient microwave absorption and infrared stealth. Nano-Micro Lett. **14**, 63 (2022). 10.1007/s40820-022-00812-w 10.1007/s40820-022-00812-w PMC8861240 35190917

[35] Y. Chang, Y. Wang, W. Wang, D. Yu, Highly efficient infrared stealth asymmetric-structure waterborne polyurethane composites prepared via one-step density-driven filler separation method. Colloids Surf. A Physicochem. Eng. Aspects **614**, 126177 (2021). 10.1016/j.colsurfa.2021.126177

[36] W. Gu, S.J.H. Ong, Y. Shen, W. Guo, Y. Fang et al., A lightweight, elastic, and thermally insulating stealth foam with high infrared-radar compatibility. Adv. Sci. **9**, e2204165 (2022). 10.1002/advs.202204165 10.1002/advs.202204165 PMC9762302 36285685

[37] Z.H. Zeng, N. Wu, J.J. Wei, Y.F. Yang, T.T. Wu et al., Porous and ultra-flexible crosslinked MXene/polyimide composites for multifunctional electromagnetic interference shielding. Nano-Micro Lett. **14**, 59 (2022). 10.1007/s40820-022-00800-0 10.1007/s40820-022-00800-0 PMC8828842 35138506

[38] Y. Xu, Z. Lin, K. Rajavel, T. Zhao, P. Zhu et al., Tailorable, lightweight and superelastic liquid metal monoliths for multifunctional electromagnetic interference shielding. Nano-Micro Lett. **14**, 29 (2021). 10.1007/s40820-021-00766-5 10.1007/s40820-021-00766-5 PMC8669089 34902083

[39] Y. Yang, N. Wu, B. Li, W. Liu, F. Pan et al., Biomimetic porous MXene sediment-based hydrogel for high-performance and multifunctional electromagnetic interference shielding. ACS Nano **16**, 15042–15052 (2022). 10.1021/acsnano.2c06164 35984219 10.1021/acsnano.2c06164

[40] T. Xue, Y. Yang, D. Yu, Q. Wali, Z. Wang et al., 3D printed integrated gradient-conductive MXene/CNT/polyimide aerogel frames for electromagnetic interference shielding with ultra-low reflection. Nano-Micro Lett. **15**, 45 (2023). 10.1007/s40820-023-01017-5 10.1007/s40820-023-01017-5 PMC9908813 36752927

[41] Q. Peng, M. Ma, Q. Chu, H. Lin, W. Tao et al., Absorption-dominated electromagnetic interference shielding composite foam based on porous and bi-conductive network structures. J. Mater. Chem. A **11**, 10857–10866 (2023). 10.1039/d3ta01369c

[42] X. Pei, G. Liu, H. Shi, R. Yu, S. Wang et al., Directional electromagnetic interference shielding of asymmetric structure based on dual-needle 3D printing. Compos. Sci. Technol. **233**, 109909 (2023). 10.1016/j.compscitech.2023.109909

[43] L. Yao, Y. Wang, J. Zhao, Y. Zhu, M. Cao, Multifunctional nanocrystalline-assembled porous hierarchical material and device for integrating microwave absorption, electromagnetic interference shielding, and energy storage. Small **19**, e2208101 (2023). 10.1002/smll.202208101 36932880 10.1002/smll.202208101

[44] Y. Luo, Y. Guo, C. Wei, J. Chen, G. Zhao et al., Lightweight, compressible, and stretchable composite foams for ultra-efficient and high-stable electromagnetic interference shielding materials. Carbon **215**, 118480 (2023). 10.1016/j.carbon.2023.118480

[45] X. Liu, Y. Li, X. Sun, W. Tang, G. Deng et al., Off/on switchable smart electromagnetic interference shielding aerogel. Matter **4**, 1735–1747 (2021). 10.1016/j.matt.2021.02.022

[46] Y. Zhang, K. Ruan, K. Zhou, J. Gu, Controlled distributed Ti_3_C_2_T_*x*_ hollow microspheres on thermally conductive polyimide composite films for excellent electromagnetic interference shielding. Adv. Mater. **35**, e2211642 (2023). 10.1002/adma.202211642 36703618 10.1002/adma.202211642

[47] Y. Dai, X. Wu, L. Li, Y. Zhang, Z. Deng et al., 3D printing of resilient, lightweight and conductive MXene/reduced graphene oxide architectures for broadband electromagnetic interference shielding. J. Mater. Chem. A **10**, 11375–11385 (2022). 10.1039/d2ta01388f

[48] M. Salari, S. Habibpour, M. Hamidinejad, S. Mohseni Taromsari, H.E. Naguib et al., Enhanced electrical properties of microcellular polymer nanocomposites *via* nanocarbon geometrical alteration: a comparison of graphene nanoribbons and their parent multiwalled carbon nanotubes. Mater. Horiz. **10**, 1392–1405 (2023). 10.1039/d2mh01303g 10.1039/d2mh01303g 36752062

[49] D. Dong, J. Ma, Z. Ma, Y. Chen, H. Zhang et al., Flexible and lightweight microcellular RGO@Pebax composites with synergistic 3D conductive channels and microcracks for piezoresistive sensors. Compos. Part A Appl. Sci. Manuf. **123**, 222–231 (2019). 10.1016/j.compositesa.2019.05.019

[50] Z. Ma, G. Zhang, Q. Yang, X. Shi, J. Li et al., Tailored morphologies and properties of high-performance microcellular poly(phenylene sulfide)/poly(ether ether ketone) (PPS/PEEK) blends. J. Supercrit. Fluids **140**, 116–128 (2018). 10.1016/j.supflu.2018.06.010

[51] L. Ma, M. Hamidinejad, L. Wei, B. Zhao, C.B. Park, Absorption-dominant EMI shielding polymer composite foams: Microstructure and geometry optimization. Mater. Today Phys. **30**, 100940 (2023). 10.1016/j.mtphys.2022.100940

[52] H. Zhang, G. Zhang, Q. Gao, M. Tang, Z. Ma et al., Multifunctional microcellular PVDF/Ni-chains composite foams with enhanced electromagnetic interference shielding and superior thermal insulation performance. Chem. Eng. J. **379**, 122304 (2020). 10.1016/j.cej.2019.122304

[53] H. Pang, L. Xu, D.-X. Yan, Z.-M. Li, Conductive polymer composites with segregated structures. Prog. Polym. Sci. **39**, 1908–1933 (2014). 10.1016/j.progpolymsci.2014.07.007

[54] Q. Huang, Z. Tang, D. Wang, S. Wu, B. Guo, Engineering segregated structures in a cross-linked elastomeric network enabled by dynamic cross-link reshuffling. ACS Macro Lett. **10**, 231–236 (2021). 10.1021/acsmacrolett.0c00852 35570780 10.1021/acsmacrolett.0c00852

[55] T. Wang, W.-W. Kong, W.-C. Yu, J.-F. Gao, K. Dai et al., A healable and mechanically enhanced composite with segregated conductive network structure for high-efficient electromagnetic interference shielding. Nano-Micro Lett. **13**, 162 (2021). 10.1007/s40820-021-00693-5 10.1007/s40820-021-00693-5 PMC8329141 34338928

[56] D. Feng, D. Xu, Q. Wang, P. Liu, Highly stretchable electromagnetic interference (EMI) shielding segregated polyurethane/carbon nanotube composites fabricated by microwave selective sintering. J. Mater. Chem. C **7**, 7938–7946 (2019). 10.1039/c9tc02311a

[57] H. Fang, W. Ye, K. Yang, K. Song, H. Wei et al., Vitrimer chemistry enables epoxy nanocomposites with mechanical robustness and integrated conductive segregated structure for high performance electromagnetic interference shielding. Compos. Part B Eng. **215**, 108782 (2021). 10.1016/j.compositesb.2021.108782

[58] J. Xu, T. Liu, Y. Zhang, Y. Zhang, K. Wu et al., Dragonfly wing-inspired architecture makes a stiff yet tough healable material. Matter **4**, 2474–2489 (2021). 10.1016/j.matt.2021.05.001

[59] D. Feng, P. Liu, Q. Wang, Selective microwave sintering to prepare multifunctional poly(ether imide) bead foams based on segregated carbon nanotube conductive network. Ind. Eng. Chem. Res. **59**, 5838–5847 (2020). 10.1021/acs.iecr.0c00090

[60] R. Sun, H.-B. Zhang, J. Liu, X. Xie, R. Yang et al., Highly conductive transition metal carbide/carbonitride(MXene)@polystyrene nanocomposites fabricated by electrostatic assembly for highly efficient electromagnetic interference shielding. Adv. Funct. Mater. **27**, 1702807 (2017). 10.1002/adfm.201702807

[61] W. Ma, W. Cai, W. Chen, P. Liu, J. Wang et al., Microwave-induced segregated composite network with MXene as interfacial solder for ultra-efficient electromagnetic interference shielding and anti-dripping. Chem. Eng. J. **425**, 131699 (2021). 10.1016/j.cej.2021.131699

[62] R.-Y. Ma, S.-Q. Yi, J. Li, J.-L. Zhang, W.-J. Sun et al., Highly efficient electromagnetic interference shielding and superior mechanical performance of carbon nanotube/polydimethylsiloxane composite with interface-reinforced segregated structure. Compos. Sci. Technol. **232**, 109874 (2023). 10.1016/j.compscitech.2022.109874

[63] G. Wang, L. Wang, L.H. Mark, V. Shaayegan, G. Wang et al., Ultralow-threshold and lightweight biodegradable porous PLA/MWCNT with segregated conductive networks for high-performance thermal insulation and electromagnetic interference shielding applications. ACS Appl. Mater. Interfaces **10**, 1195–1203 (2018). 10.1021/acsami.7b14111 29206437 10.1021/acsami.7b14111

[64] Z. Ma, A. Wei, Y. Li, L. Shao, H. Zhang et al., Lightweight, flexible and highly sensitive segregated microcellular nanocomposite piezoresistive sensors for human motion detection. Compos. Sci. Technol. **203**, 108571 (2021). 10.1016/j.compscitech.2020.108571

[65] Y. Wu, Y. Zhao, M. Zhou, S. Tan, R. Peymanfar et al., Ultrabroad microwave absorption ability and infrared stealth property of nano-micro CuS@rGO lightweight aerogels. Nano-Micro Lett. **14**, 171 (2022). 10.1007/s40820-022-00906-5 10.1007/s40820-022-00906-5 PMC9392679 35987861

[66] L. Li, M. Shi, X. Liu, X. Jin, Y. Cao et al., Ultrathin titanium carbide (MXene) films for high-temperature thermal camouflage. Adv. Funct. Mater. **31**, 2101381 (2021). 10.1002/adfm.202101381

[67] Z. Deng, P. Jiang, Z. Wang, L. Xu, Z.-Z. Yu et al., Scalable production of catecholamine-densified MXene coatings for electromagnetic shielding and infrared stealth. Small **19**, e2304278 (2023). 10.1002/smll.202304278 37431209 10.1002/smll.202304278

[68] W. Ma, W. Cai, W. Chen, P. Liu, J. Wang et al., A novel structural design of shielding capsule to prepare high-performance and self-healing MXene-based sponge for ultra-efficient electromagnetic interference shielding. Chem. Eng. J. **426**, 130729 (2021). 10.1016/j.cej.2021.130729

[69] F. Pan, Y. Shi, Y. Yang, H. Guo, L. Li et al., *Porifera*-inspired lightweight, thin, wrinkle-resistance, and multifunctional MXene foam. Adv. Mater. **36**, e2311135 (2024). 10.1002/adma.202311135 38146773 10.1002/adma.202311135

[70] Z. Jiao, W. Huyan, F. Yang, J. Yao, R. Tan et al., Achieving ultra-wideband and elevated temperature electromagnetic wave absorption via constructing lightweight porous rigid structure. Nano-Micro Lett. **14**, 173 (2022). 10.1007/s40820-022-00904-7 10.1007/s40820-022-00904-7 PMC9399338 35999287

