# Supplementary material for: Lightweight Dual-Functional Segregated Nanocomposite Foams for Integrated Infrared Stealth and Absorption-Dominant Electromagnetic Interference Shielding
Source: Nanomicro Lett. 2024 Jun 17;16:223. doi: 10.1007/s40820-024-01450-0 (PMC11183016; doi:10.1007/s40820-024-01450-0)
Supplement: Supplementary file 1 — Supplementary file1 (PDF 1256 KB) [file 40820_2024_1450_MOESM1_ESM.pdf]

Supplementary Information for

Lightweight Dual-Functional Segregated Nanocomposite Foams for Integrated Infrared Stealth and Absorption-Dominant Electromagnetic Interference Shielding

Zhonglei Ma[1, 4, *], Ruochu Jiang[1, 4], Jiayao Jing[2], Songlei Kang[2], Li Ma[3], Kefan Zhang[1, 4], Junxian Li[1], Yu Zhang[1], Jianbin Qin[1, 4], Shuhuan Yun[1], Guangcheng Zhang[1, 4, *]

[1]MOE Key Laboratory of Material Physics and Chemistry under Extraordinary Conditions, Shaanxi Key Laboratory of Macromolecular Science and Technology, School of Chemistry and Chemical Engineering, Northwestern Polytechnical University, Xi'an, 710072, P. R. China

[2]College of Chemistry and Chemical Engineering, Key Laboratory of Auxiliary Chemistry and Technology for Chemical Industry, Ministry of Education, Shaanxi University of Science and Technology, Xi'an, 710021, P. R. China

[3]Department of Mechanical and Industrial Engineering, University of Toronto, 5 King's College Road, Toronto, Ontario M5S 3G8, Canada

[4]Chongqing Innovation Center, Northwestern Polytechnical University, Chongqing, 401135, P. R. China

*Corresponding authors. E-mail: mazl@nwpu.edu.cn (Zhonglei Ma); zhangguc@nwpu.edu.cn (Guangcheng Zhang)

S1 Gas saturation behaviors of TPAE beads with different shore D hardnesses

The gas saturation behaviors of commercial TPAE beads with different types of Pebax 3533, 5533, and 7033 (their shore D hardnesses are 35, 55, and 70 respectively) are evaluated in an autoclave containing supercritical CO_2 (SC-CO_2) at 45 °C and 15 MPa. Figure S1 shows the effect of saturation time on the gas concentration in TPAE beads. As can be seen, the gas concentration in TPAE beads increases rapidly at the beginning, and then gradually reaches the equilibrium with the increasing saturation time. At the saturation time of 5 h, the TPAE beads with different shore D hardnesses of 35, 55 and 70 all achieve the completely saturated gas concentrations of 135, 78, and 43 mg CO_2 per

gram of TPAE respectively. With higher shore D hardness, the TPAE beads exhibit the decreased gas concentration due to the higher crystallinity as the molecular chains contain more crystalline polyamide hard segments and less polyether soft segments.

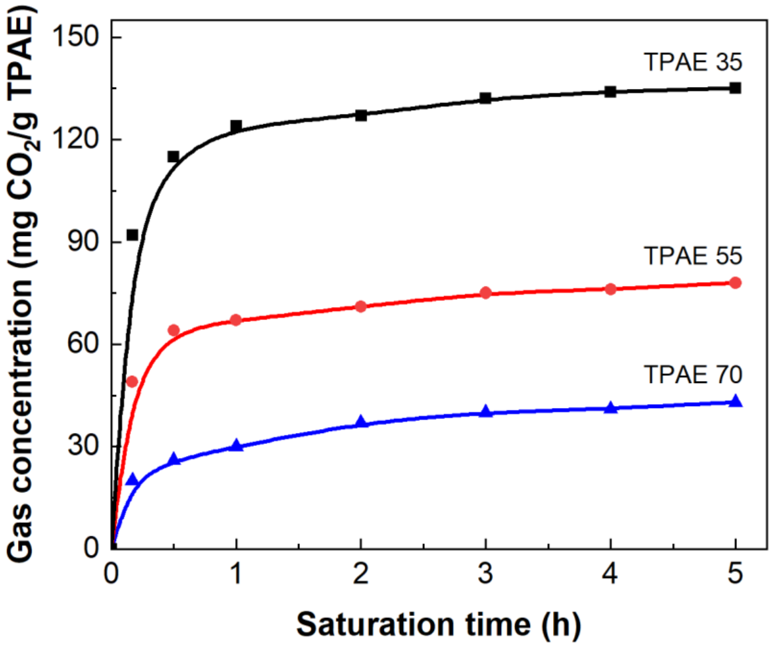

Fig. S1 Effect of saturation time on the gas concentration in TPAE beads with different shore D hardnesses at 15 MPa and 45 °C

S2 Microcellular foaming of TPAE beads with different shore D hardnesses

The SC-CO_2 foaming abilities of TPAE beads with different shore D hardnesses of 35, 55 and 70 are evaluated. As shown in Fig. S2, the TPAE beads with a low shore D hardness of 35 exhibit the excellent foamability with tailorable microcellular structures. In comparison, the TPAE beads with higher shore D hardnesses of 55 and 70 exhibit poor foamabilities with few or no obvious microcellular structures. This is mainly because that the TPAE beads with higher shore D hardnesses contain more crystalline polyamide hard segments and less polyether soft segments in molecular chains, resulting in the higher crystallinities, decreased gas concentration and thus poor foamabilities. The increased crystallinities with the higher shore D hardnesses can be demonstrated by the DSC analysis (Fig. S3), which show much larger melting enthalpy during heating. Therefore, the TPAE beads with a relatively low shore D hardnesses of 35, which show the relatively higher saturated gas concentration and foamability, are used for the fabrication of lightweight and high-performance segregated nanocomposite foams.

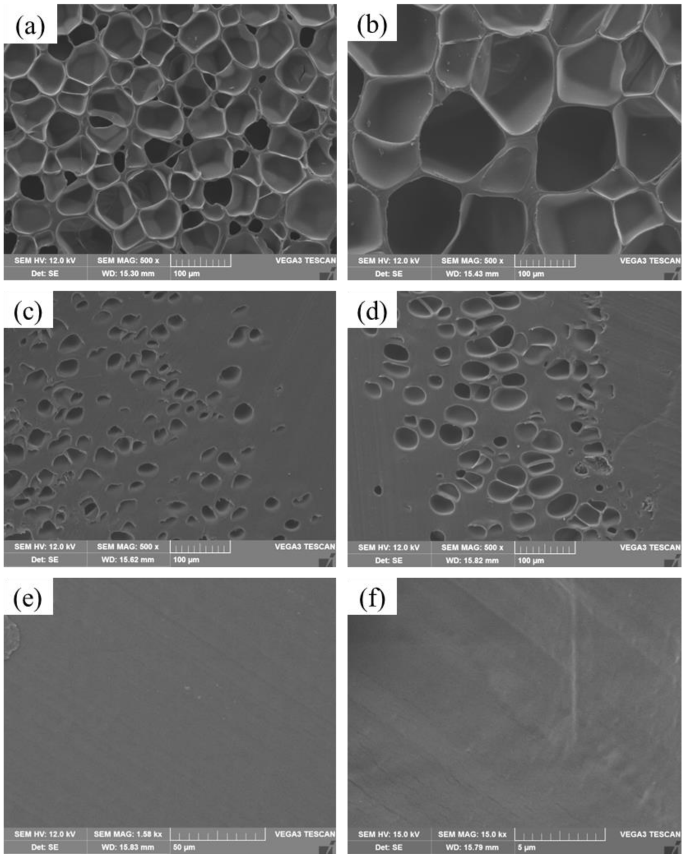

Fig. S2 SEM images of the microcellular foamed TPAE beads with different shore D hardnesses of **a, b** 35, **c, d** 55 and **e, f** 70 at 125 °C for 30 and 120 s, respectively

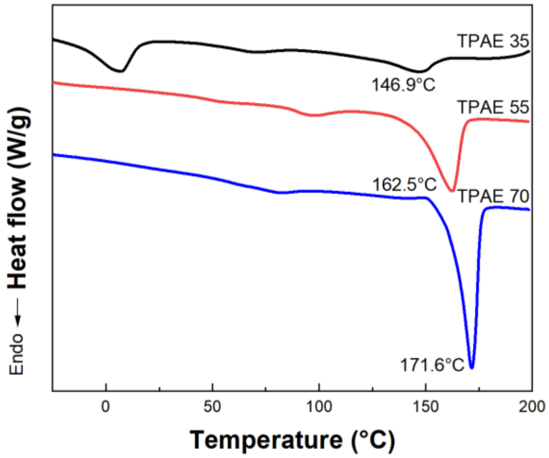

Fig. S3 DSC thermograms of the TPAE beads with different shore D hardnesses of 35, 55 and 70

S3 $Ti_3C_2T_x$ content of segregated nanocomposite foams

The $Ti_3C_2T_x$ content, expressed as volume fraction of $Ti_3C_2T_x$ MXene, is calculated by:

$$\mathrm{vol\%}\left(\mathrm{Ti_3C_2T_x}\right)=\frac{V_{\mathrm{Ti_3C_2T_x}}}{V_\mathrm{c}}\times 100\%=\frac{m_{\mathrm{Ti_3C_2T_x}}}{\rho_{\mathrm{Ti_3C_2T_x}}\cdot V_\mathrm{c}}\times 100\%$$

where $V_{\mathrm{Ti_3C_2T_x}}$ and V_c are the volumes of $Ti_3C_2T_x$ MXene and segregated nanocomposite foams, respectively. V_c is the volume of segregated nanocomposite foams. $m_{\mathrm{Ti_3C_2T_x}}$ is the mass of $Ti_3C_2T_x$ MXene assembled on the microcellular TPAE beads. $\rho_{\mathrm{Ti_3C_2T_x}}$ is the mass density (3.2 g/cm^3) of $Ti_3C_2T_x$ MXene. The calculated $Ti_3C_2T_x$ contents of segregated nanocomposite foams with three different expansion ratios are presented in Table S1. The microcellular TPAE@$Ti_3C_2T_x$ beads are obtained by dip-coating the microcellular TPAE beads in $Ti_3C_2T_x$ aqueous dispersion with concentrations of 5, 10, 15 and 20 mg/mL.

Table S1 $Ti_3C_2T_x$ contents of the segregated nanocomposite foams

**Expansion ratio**	**$Ti_3C_2T_x$ dispersion concentration (mg/mL)**	**$Ti_3C_2T_x$ content (vol%)**
2.5	5	0.6
	10	1.3
	15	1.6
	20	2.5
4.2	5	0.5
	10	1.0
	15	1.3
	20	1.7
5.5	5	0.5
	10	1.1
	15	0.9
	20	1.3

S4 Digital images of solid and microcellular TPAE beads

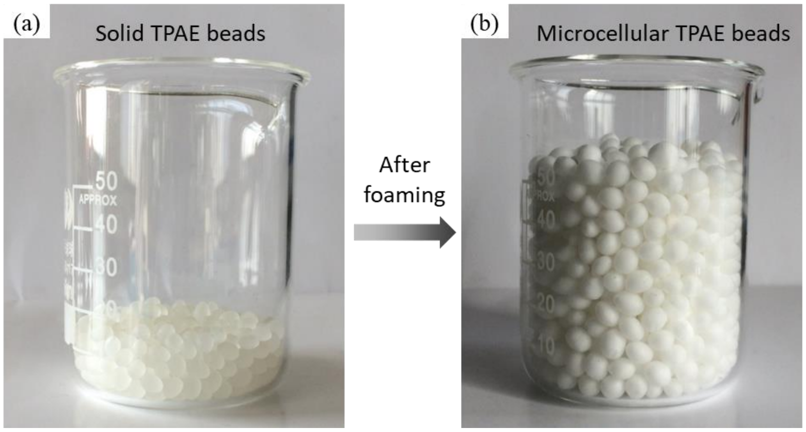

Fig. S4 Digital images of the **a** solid and **b** microcellular TPAE beads

S5 Digital images of microcellular TPAE beads with different expansion ratios

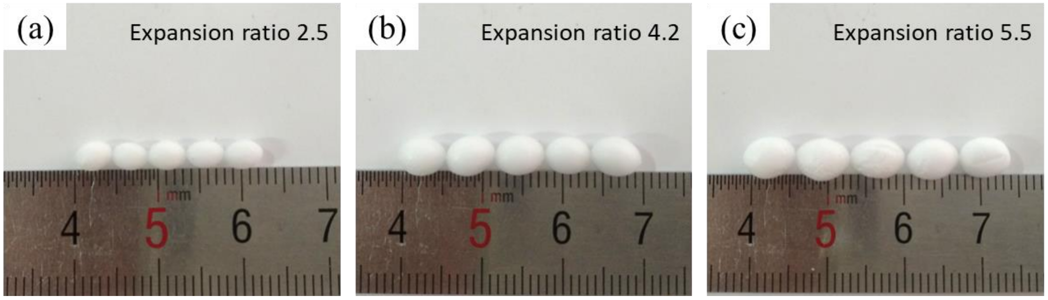

Fig. S5 Digital images of the microcellular TPAE beads with different expansion ratios of 2.5, 4.2 and 5.5

S6 Schematic diagrams for adhesion interfaces between microcellular TPAE beads

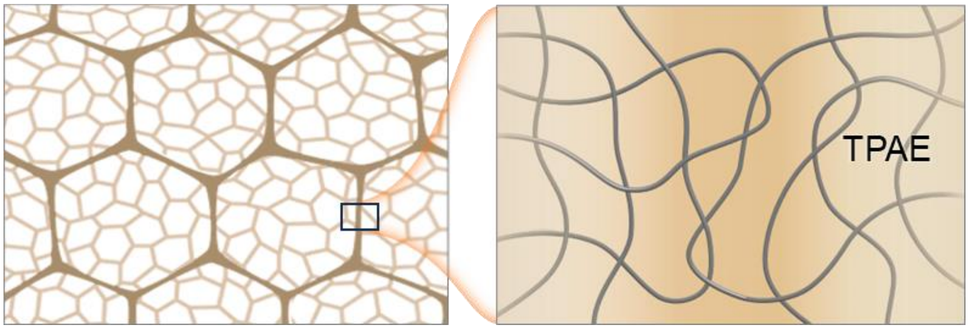

Fig. S6 Schematic diagrams for adhesion interfaces between adjacent microcellular TPAE beads

S7 Digital image of microcellular TPAE foams

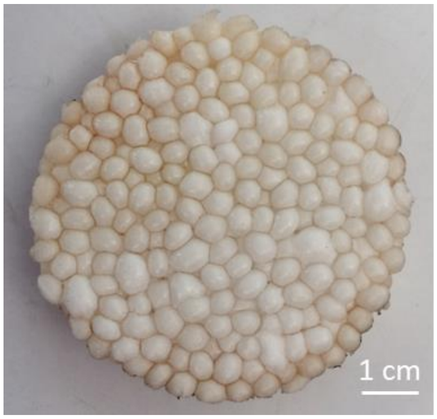

Fig. S7 Digital image of the microcellular TPAE foams

S8 SEM image of Ti_3AlC_2 (MAX)

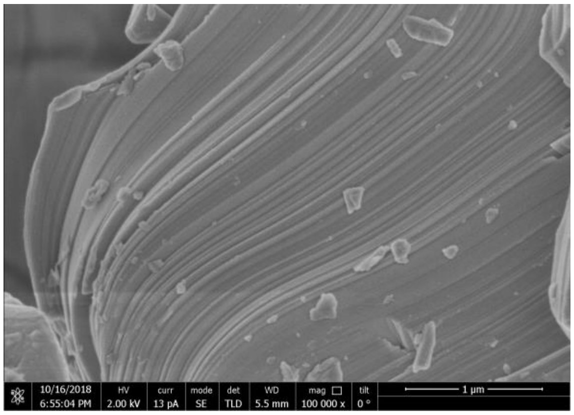

Fig. S8 SEM image of Ti_3AlC_2 (MAX)

S9 Digital image of microcellular TPAE@$Ti_3C_2T_x$ beads

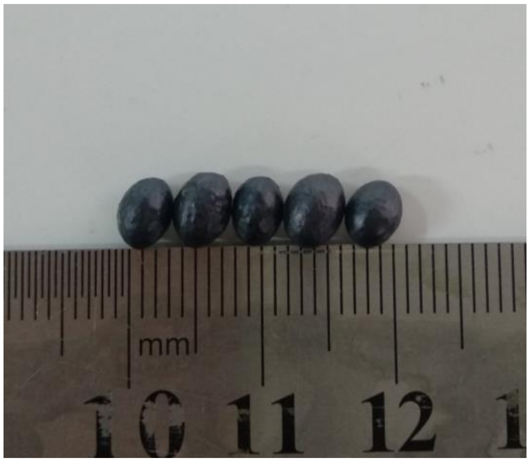

Fig. S9 Digital image of the microcellular TPAE@$Ti_3C_2T_x$ beads

S10 Mass densities of microcellular TPAE foams and segregated nanocomposite foams

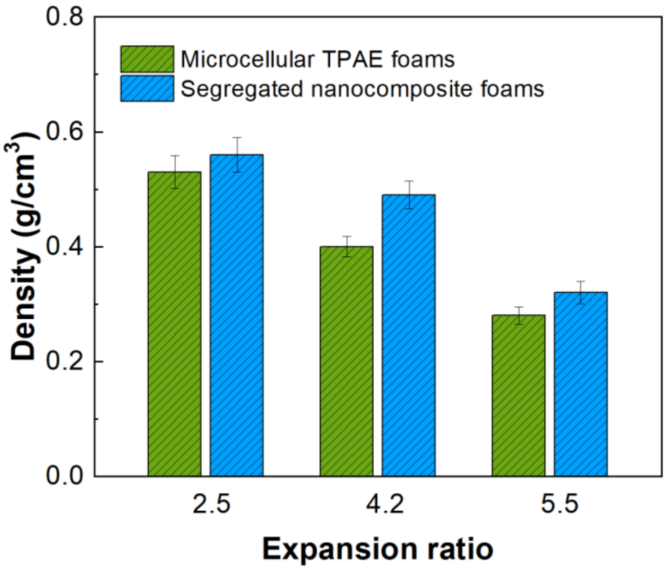

Fig. S10 Mass densities of the microcellular TPAE foams and segregated nanocomposite foams

S11 Lightweight microcellular TPAE foams and segregated nanocomposite foams floated on the water

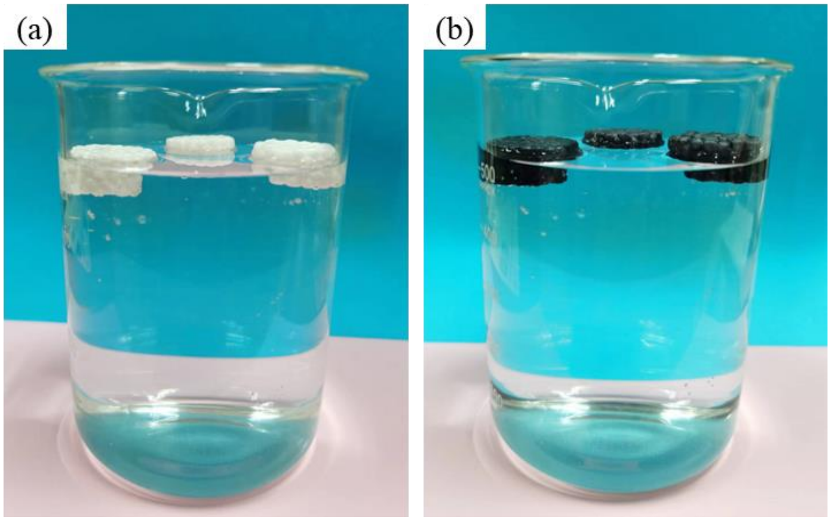

Fig. S11 Lightweight **a** microcellular TPAE foams and **b** segregated nanocomposite foams floated on the water.

S12 Radiation temperatures of microcellular TPAE foams and segregated nanocomposite foams

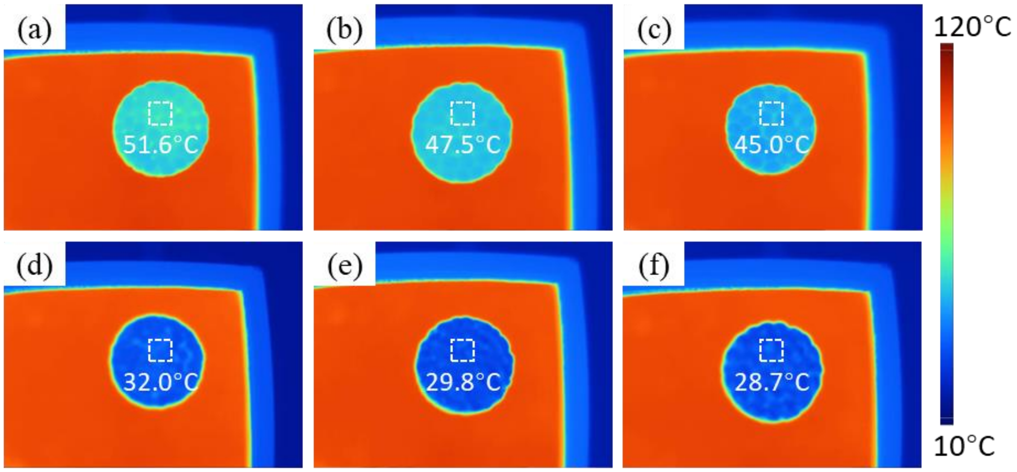

Fig. S12 Radiation temperatures of the microcellular TPAE foams with different expansions of **a** 2.5, **b** 4.2 and **c** 5.5, respectively. Radiation temperatures of the segregated nanocomposite foams with different expansion ratios of **d** 2.5, **e** 4.2 and **f** 5.5

S13 Infrared stealth performances of microcellular TPAE foams and segregated nanocomposite foams

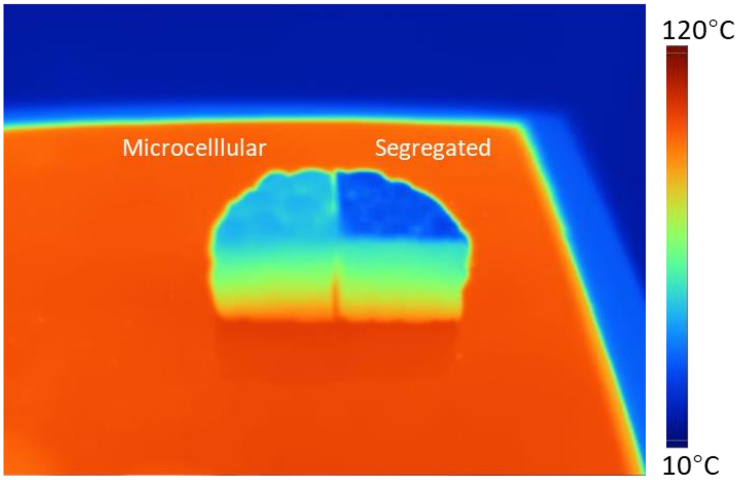

Fig. S13 Radiation temperatures of the fracture surfaces of microcellular TPAE foams and segregated nanocomposite foams with the infrared object temperature of 100 °C

S14 Comparation of infrared stealth performances with other cellular materials

The infrared stealth performances of segregated nanocomposite foams are compared with other commercial polymeric cellular materials such as polyurethane (PU) foams,

polystyrene (PS) foams and polypropylene (PP) foams with the same thickness. As shown in Fig. S14, the other polymeric cellular materials all present higher radiation temperatures than the segregated nanocomposite foams, indicating the superior infrared stealth performances of segregated nanocomposite foams.

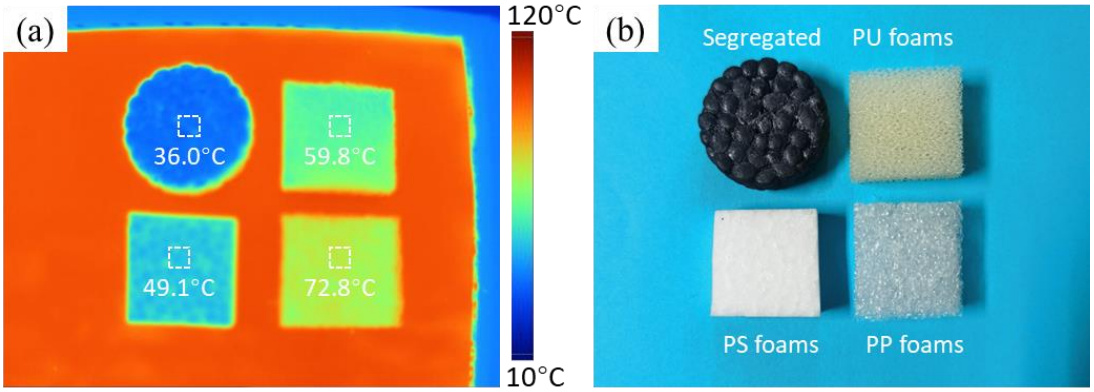

Fig. S14 Radiation temperatures of the segregated nanocomposite foams and other commercial polymeric foams with the same infrared object temperature of 100 °C

S15 Time-dependent compression stress of segregated nanocomposite foams upon repeated compression strains

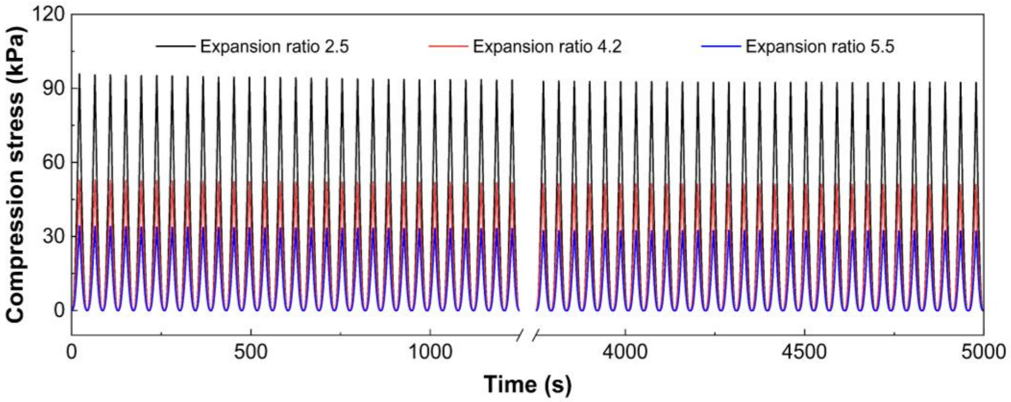

Fig. S15 Time-dependent compression stress of the segregated nanocomposite foams upon repeated compression strains of 25%

S16 Tensile properties of the segregated nanocomposite foams with an expansion ratio of 4.2

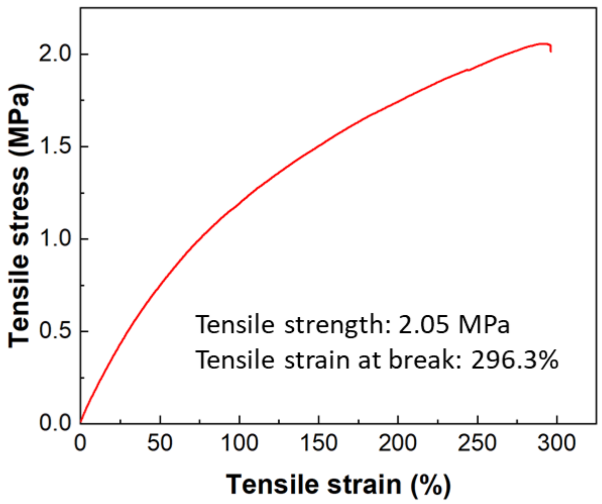

Fig. S16 Tensile stress-strain curve of the segregated nanocomposite foams with an expansion ratio of 4.2 at the speed of 5 mm/min.

S17 Electrical conductivities of segregated nanocomposite foams

Figure S17 shows the electrical properties of segregated nanocomposite foams with three different expansion ratios of 2.5, 4.2 and 5.5. The segregated nanocomposite foams with larger microcellular TPAE bead expansion ratio exhibit decreased electrical conductivity with the same $Ti_3C_2T_x$ dispersion concentration during dip-coating. This is attributed that the microcellular TPAE beads with smaller expansion ratio possess higher specific surface area, resulting in the higher $Ti_3C_2T_x$ content and relatively more efficient 3D conductive networks in the segregated nanocomposite foams. With higher $Ti_3C_2T_x$ dispersion concentration, the segregated nanocomposite foams exhibit larger electrical conductivity due to the more $Ti_3C_2T_x$ MXene assembled on the surfaces of microcellular TPAE beads. With the same $Ti_3C_2T_x$ dispersion concentration of 20 mg/mL during dip-coating, for instance, the obtained segregated nanocomposite foams with expansion ratios of 2.5, 4.2 and 5.5 show electrical conductivities of 0.57, 0.23 and 0.09 S/m at the ultralow $Ti_3C_2T_x$ contents of 2.5, 1.7 and 1.3 vol%, respectively.

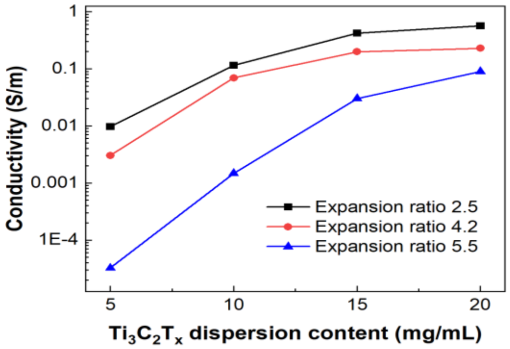

Fig. S17 Electrical conductivities of the segregated nanocomposite foams

S18 EDS mapping images of the segregated nanocomposite foams

The EDS mapping images in Fig. S18 indicate that the segregated nanocomposite foams possess relatively integrated conductive paths with different expansion ratios. This demonstrates the successful construction of conductive segregated networks at ultralow $Ti_3C_2T_x$ contents, which is crucial to the EMI shielding performances of the segregated nanocomposite foams.

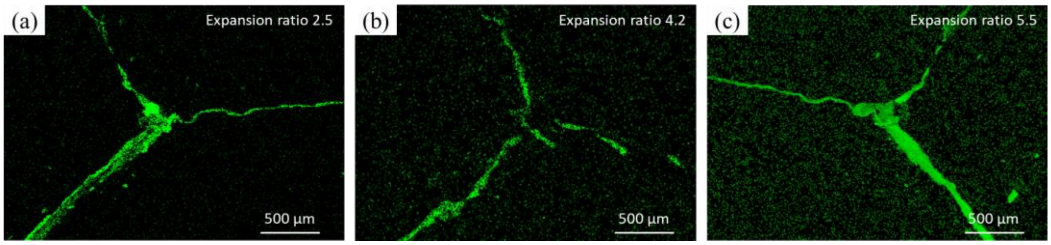

Fig. S18 EDS mapping images of the segregated nanocomposite foams with different expansion ratios

S19 *A*, *R* and *T* coefficients of segregated nanocomposite foams

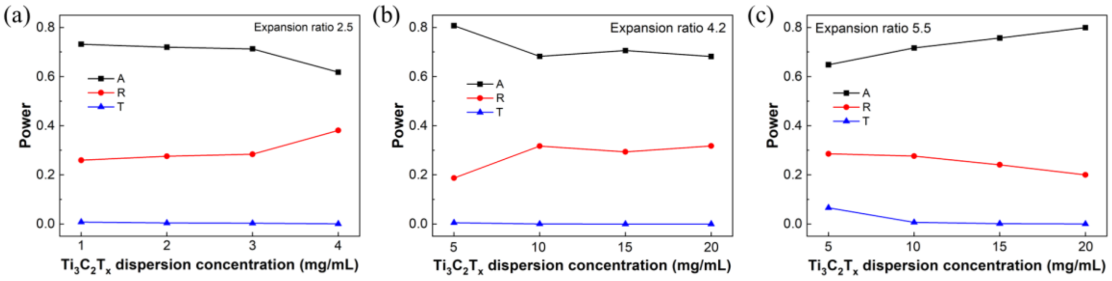

Fig. S19 *A*, *R* and *T* coefficients of the segregated nanocomposite foams with different expansion ratios of **a** 2.5, **b** 4.2 and **c** 5.5

S20 Long-term infrared stealth and EMI shielding stabilities of segregated nanocomposite foams

The long-term infrared stealth and EMI shielding stabilities of segregated nanocomposite foams were evaluated after being placed in the air for 2 months. As shown in Fig. S20a, the infrared emissivity of segregated nanocomposite foams is slightly increased from 0.13 to 0.28 due to the partial oxidation of $Ti_3C_2T_x$ MXene. Nevertheless, the infrared emissivity still remains at a low value, which can satisfactorily meet the requirements in infrared stealth applications. On the hot stage with a high temperature of 100°C, the segregated nanocomposite foams after 2 months exhibit a slightly increased radiation temperature of 36.8°C with an increase of only 3.6°C. This is mainly attributed to the highly-effective thermal insulation of microcellular structures and low infrared emissivity of slightly oxidized $Ti_3C_2T_x$ MXene on the surfaces. Fig. S20b shows that the segregated nanocomposite foams exhibit a slightly decreased EMI SE of 39.7 dB with a small reduction of only 4.3 dB after being placed in the air for 2 months, benefitting from the stable interior $Ti_3C_2T_x$ conductive networks protected by TPAE. The results indicate that the segregated nanocomposite foams possess certain long-term infrared stealth and EMI shielding stabilities in the air environment.

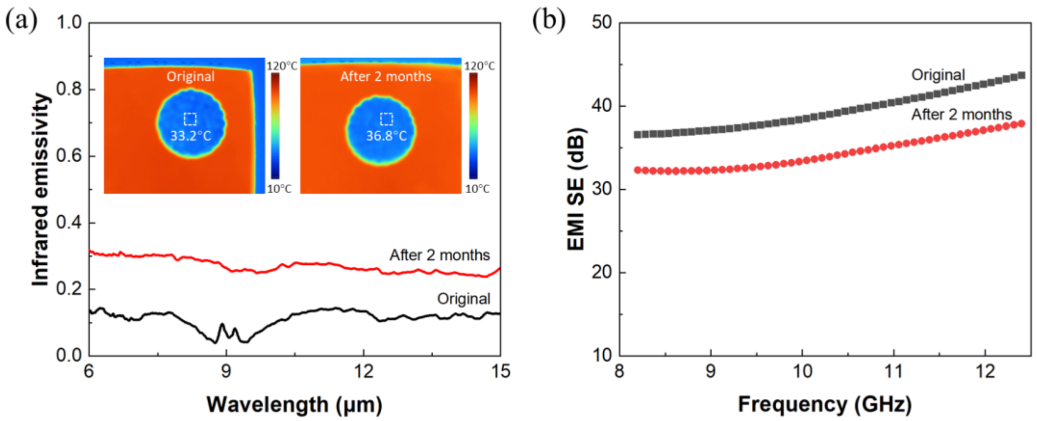

Fig. S20 a Infrared emissivity and radiation temperatures (insets) of the segregated nanocomposite foams with an expansion ratio of 4.2 after 2 months. **b** EMI shielding performances of the segregated nanocomposite foams with an expansion ratio of 4.2 after 2 months
